# Increased locomotor activity via regulation of GABAergic signalling in *foxp2* mutant zebrafish—implications for neurodevelopmental disorders

**DOI:** 10.1038/s41398-021-01651-w

**Published:** 2021-10-14

**Authors:** Teresa M. Lüffe, Andrea D’Orazio, Moritz Bauer, Zoi Gioga, Victoria Schoeffler, Klaus-Peter Lesch, Marcel Romanos, Carsten Drepper, Christina Lillesaar

**Affiliations:** 1 Center of Mental Health, Department of Child and Adolescent Psychiatry, University Hospital of Würzburg, Würzburg, Germany; 2 Center of Mental Health, Division of Molecular Psychiatry, University of Würzburg, Würzburg, Germany; 3 Laboratory of Psychiatric Neurobiology, Institute of Molecular Medicine, I.M. Sechenov First Moscow State Medical University, Moscow, Russia; 4 Department of Neuroscience, School for Mental Health and Neuroscience (MHeNS), Maastricht University, Maastricht, The Netherlands

**Keywords:** Molecular neuroscience, Comparative genomics

## Abstract

Recent advances in the genetics of neurodevelopmental disorders (NDDs) have identified the transcription factor *FOXP2* as one of numerous risk genes, e.g. in autism spectrum disorders (ASD) and attention-deficit/hyperactivity disorder (ADHD). FOXP2 function is suggested to be involved in GABAergic signalling and numerous studies demonstrate that GABAergic function is altered in NDDs, thus disrupting the excitation/inhibition balance. Interestingly, GABAergic signalling components, including glutamate-decarboxylase 1 (Gad1) and GABA receptors, are putative transcriptional targets of FOXP2. However, the specific role of FOXP2 in the pathomechanism of NDDs remains elusive. Here we test the hypothesis that Foxp2 affects behavioural dimensions via GABAergic signalling using zebrafish as model organism. We demonstrate that *foxp2* is expressed by a subset of GABAergic neurons located in brain regions involved in motor functions, including the subpallium, posterior tuberculum, thalamus and medulla oblongata. Using CRISPR/Cas9 gene-editing we generated a novel *foxp2* zebrafish loss-of-function mutant that exhibits increased locomotor activity. Further, genetic and/or pharmacological disruption of Gad1 or GABA-A receptors causes increased locomotor activity, resembling the phenotype of *foxp2* mutants. Application of muscimol, a GABA-A receptor agonist, rescues the hyperactive phenotype induced by the *foxp2* loss-of-function. By reverse translation of the therapeutic effect on hyperactive behaviour exerted by methylphenidate, we note that application of methylphenidate evokes different responses in wildtype compared to *foxp2* or *gad1b* loss-of-function animals. Together, our findings support the hypothesis that *foxp2* regulates locomotor activity via GABAergic signalling. This provides one targetable mechanism, which may contribute to behavioural phenotypes commonly observed in NDDs.

## Introduction

Ever larger and improved genetic studies have enabled substantial progress in the discovery of risk genes for neurodevelopmental disorders (NDDs), such as autism spectrum disorders (ASD) and attention-deficit/hyperactivity disorder (ADHD). However, the involvement of most of the identified risk genes in the pathomechanism remains elusive and requires further extensive functional investigations. Recently, a genome-wide association study of ADHD highlighted 12 loci with genome-wide significance [1]. One of the loci, located on chromosome 7, harbours the gene *FOXP2*, whose possible role in NDDs is substantiated by several studies [2, 3].

FOXP2 belongs to the P subfamily of FOX transcription factors, which are recognised by a highly conserved DNA-binding motif termed winged helix or forkhead domain and by additional subfamily-characteristic domains. It possesses domains involved in homo- and/or hetero-dimerisation, primarily with FOXP1 and FOXP4, as well as in interactions with other proteins, which allows both transcriptional repression and activation in a context-dependent manner [4, 5].

In human, *FOXP2* is predominantly expressed in the central nervous system (CNS) with prominent presence in the deep layers of the developing cortical plate, the basal ganglia, the thalamus, the inferior olive and the cerebellum [6]. Apart from humans, *Foxp2*/*foxp2* expression has been described for additional mammalian species as well as for birds, reptiles, amphibians and fish, including zebrafish (*Danio rerio)* [7–17]. These studies revealed a highly concordant localisation within the CNS, which coincides with regions involved in motor functions and therefore suggest a functional conservation of Foxp2. The CNS expression of *Foxp2/foxp2* starts during embryonic development and continues postnatally into adulthood and thus indicates a role both in development and maintenance of the CNS. Accordingly, critical functions of Foxp2 in cell differentiation, neurite outgrowth, dendrite morphogenesis, axon guidance and synaptic plasticity have been demonstrated [18–28].

The biological pathways underlying Foxp2 deficiency-induced behavioural phenotypes are still largely unknown. However, experimental evidence indicates that altered GABAergic signalling may play a pivotal role in this context [23, 29]. Thus, *Foxp2* is expressed by striatal medium spiny neurons (MSNs) [28], a class of GABAergic neurons that accounts for the majority of the neurons in striatum. Upon *Foxp2* impairment, the neurite morphology of these neurons is altered [19, 28]. Further, in *Foxp2* loss-of-function mice, an increased inhibitory presynaptic strength of the striatal direct pathway MSNs due to increased GABA release was observed [29]. This phenotype was accompanied by increased expression of *Glutamate-decarboxylase 1* (*Gad1*, also known as *Gad67*). In addition, Foxp2 transcriptional targets include genes involved in GABAergic neurotransmission, such as *Gad1*, *Gad2* and GABA receptor subunits [5, 28].

Neuronal network activity, such as in the cortico-striatal circuitry, is controlled by a tightly regulated interaction between excitation (E) and inhibition (I). Under physiological conditions, a definite E/I ratio is maintained via balanced synaptic communication between glutamatergic and GABAergic neurons [30, 31], resulting in the so-called ‘E/I balance’. Emerging evidence indicates that the E/I balance is dysregulated in NDDs, including ADHD and ASD [32–35]. GABAergic signalling impacts on the developing brain through a variety of regulatory mechanisms ranging from myelination to synaptic communications [36, 37]. These mechanisms program the maturation of interneurons, e.g. MSN, thus allowing strong GABA-mediated feed-forward inhibition to maintain the stability of local networks. The relevance of compromised GABA-mediated inhibition for the aetiology of NDDs is supported by multiple findings both in humans and in model organisms. In human, variants of genes encoding for GABAergic signalling components are associated with ADHD [38–41], and mice with genetic disruption of GABAergic signalling components mirror core ADHD symptoms [42–45]. Further, structural brain imaging in ADHD patients shows alterations in regions with prominent GABAergic neuron populations, such as the striatum [46]. Moreover, magnetic resonance spectroscopy revealed altered GABA levels in specific brain regions, including motor cortices, striatum and thalamus of ADHD patients [47–50].

In the present study, we explore the hypothesis that *foxp2* regulates behavioural activity via GABAergic signalling. Considering that genetic variants of *FOXP2* and/or genes encoding for GABAergic signalling components are associated with ADHD, ASD as well as speech and language disorders [1–3, 38–41], our findings may have implications for NDDs.

## Materials and methods

For detailed descriptions see Supplementary Materials and Methods.

### Fish husbandry and embryo preparation

Experiments were performed on the AB/AB wildtype zebrafish strain (zfin id.: ZDB-GENO-960809-7). Animal handling was performed in accordance with the regulations for animal welfare of the District Government of Lower Franconia, Germany. Larvae were raised in Danieau’s solution with or without methylene blue (Cold Spring Harb. Protoc., 2011) at 28 °C with a light/dark cycle of 14/10 h. Determination of developmental stage was according to [51].

### Whole-mount RNA in situ hybridisation, immunohistochemistry and image acquisition

Embryos were raised in Danieau’s solution containing 0.2 mM 1-phenyl-2-thiourea to suppress pigmentation, manually dechorionated and fixed in 4% paraformaldehyde (PFA) in phosphate-buffered saline (PBS). A cDNA template for *foxp2* covering the last three exons, including the 3′UTR was cloned and used for in vitro transcription of a DIG-labelled RNA ISH probe (for primers, see Table S1). Whole-mount RNA ISH was performed in accordance with [52]. For two-colour RNA ISH, a mix of DIG-labelled *foxp2* and FLUO-labelled *gad1a* (previously *gad67a*) RNA probes was applied, and the RNA hybrids were visualised with NBT/BCIP or Fast red, respectively. To further display co-localisation of *foxp2* and *gad1a* double-stained embryos were cryo-sectioned.

To visualise major neurite bundles and tracts, fixed embryos were immunostained with anti-acetylated tubulin (AcTub). Apoptotic cells were identified by application of anti-cleaved caspase 3 (cCasp3). Alexa Fluor 488-conjugated secondary antibodies were used for detection of both primary antibodies. Light and fluorescence images were acquired using a Zeiss Axiophot light microscope, a Leica M205 FA fluorescence microscope or a Zeiss LSM 780 confocal microscope and further processed and analysed using Image J with appropriate plug-ins.

### *foxp2* and *gad1b* loss-of-function

**Fig. 1:**
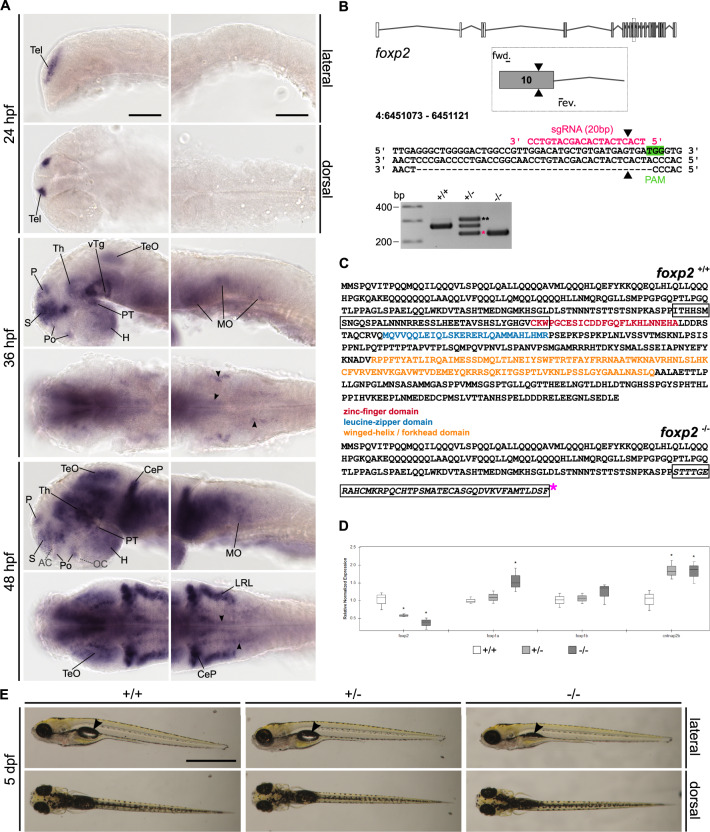
Expression of *foxp2* in the developing CNS of zebrafish and generation of a *foxp2* knock-out line using CRISPR/Cas9. **A** Whole-mount RNA in situ hybridisation (ISH) in the developing zebrafish. Anterior is to the left. Abbreviations are listed in Table S4. Arrowheads indicate distinct *foxp2*-positive populations in the MO. Scale bars: 100 µm. **B** *foxp2* exon–intron structure with coding (grey) and non-coding exons (white). The sgRNA (pink) targeting *foxp2* exon 10 and the primer binding sites for genotyping PCR are indicated. The CRISPR/Cas9-induced double-strand break (black triangle) caused a 40 bp deletion represented by a PCR product of 228 bp (pink asterisk) in *foxp2*^+/^^−^ and *foxp2*^−/−^. A third band of ~320 bp corresponds a heterodimer of wildtype and mutated PCR product (black asterisks). **C** Predicted amino acid sequence of the wildtype (top) and mutated allele (bottom). The frameshifted sequence (black box) is interrupted by a premature stop codon (pink asterisk) N-terminal of the zinc-finger domain. **D** Relative normalised expression of *foxp2, foxp1a, foxp1b* and *cntnap2b* in *foxp2*^+/+^ (white), *foxp2*^+/−^ (light grey) and *foxp2*^−/−^ (dark grey) based on qPCR. *P < 0.05. **E** Live images of 5 dpf old *foxp2*^+/+^, *foxp2*^+/−^ and *foxp2*^−/−^. Anterior is to the left. *foxp2*^−/−^ show alterations in the development/inflation of the swim bladder (black arrow) in 20% of the cases. Scale bar, 1 mm.

To create a *foxp2* loss-of-function, we applied the CRISPR/Cas9 gene-editing tool and injected a cocktail of a sgRNA targeting exon 10 of *foxp2* (Fig. 1B, Table S1) and the Cas9 protein into the animal pole of fertilised one-cell stage eggs. Injected F_0_ embryos were raised and tested for germline transmission of indel mutations by outcrossing with AB/AB wildtypes and genotyping the offspring. The F_1_ generation was generated by outcrossing positive F_0_ fish with AB/AB wildtypes and used to characterise the indel mutations by Sanger sequencing. Finally, the deletion mutation described in Fig. 1 was selected for subsequent investigations. For the experiments described below, F_2_ or F_3_ embryos and larvae generated by outcrosses of *foxp2*^*+/−*^ to AB/AB or intercrosses of *foxp2*^*+/*^^−^ were used.

A *gad1b* loss-of-function was created by injections of a splice-inhibiting morpholino oligonucleotide (GeneTools, Table S1) into the animal pole of fertilised one-cell stage eggs. Aberrant splicing was confirmed by RT-PCR and Sanger sequencing at 1 and 5 days post fertilisation (dpf).

### Quantitative real-time PCR (qPCR)

Quantitative real-time RT-PCR (qPCR) was performed on 5 dpf old *foxp2*^*−/−*^, *foxp2*^*+/*^^−^ and *foxp2*^*+/+*^ siblings. The tail of each embryo was cut for gDNA extraction and subsequent genotyping. The remaining tissue was used for total RNA extraction and subsequent cDNA synthesis. RNA from 10 pooled embryos was isolated for each genotype, and each target gene (Table S2) was represented by a triplet (technical replicates) of each biological sample (*n* = 3) for each genotype. No RT control (NRT) and no template control (NTC) were included as negative controls. Final quantification and calculation were conducted with the comparative C_t_ (2-ΔΔCt) method using *actb1* and *gapdh* as housekeeping genes (Table S2). Significant group differences were determined by applying a one-way ANOVA with the significance level set to 0.05.

### Locomotor tracking

Locomotion in 5 dpf old larvae was tracked by the semi-automatic system ZebraBox and the commercial software ZebraLab (View Point). Larvae were placed in individual wells of a 12-well plate containing 1 ml of Danieau’s solution. The surrounding water was kept constant at 28 °C. Swimming tracks were recorded in the dark with an infrared backlight with a wavelength of 850 nm by an integrated infrared camera with 30 fps. Three activity levels were defined by the following thresholds: inactive, <0.2 cm/s; low activity, >0.2 cm/s and <1 cm/s; high activity, >1 cm/s. Unless stated differently below, larvae were tracked for a total duration of 10 min, with a 5 min habituation and a 5 min test phase. Any larvae exhibiting severe morphological malformations were excluded from the behaviour analysis, and individual wells where misstracking due to technical issues was observed were excluded from the subsequent data analysis. Both exclusion criteria were applied before knowing about the genotype. For final group comparison, only data collected during the test phase was considered for analysis. Locomotor activity was determined by four different parameters: total distance swum, mean velocity during low/high activity or both (total mean velocity), duration of inactivity, low or high activity and the number of events in inactive, low and high activity phase.

### Pharmacological treatments

All substances are listed in Table S3. Glutamate-decarboxylase (Gad) activity was inhibited by the Gad antagonist L-allylglycine (Santa Cruz Biotechnology). A 1 M L-allylglycine stock solution was further diluted in Danieau’s solution to a working concentration of 200 mM. After tracking 5 dpf old wildtype larvae for 10 min without treatment (Pre) as described above, 500 µl of the total 1 ml Danieau’s solution was replaced by the L-allylglycine working solution (final concentration of 100 mM) or by Danieau’s solution without any added substance. After an incubation period of 1 h, the locomotor activity was recorded once per h for 10 min with 5 min habituation and 5 min test periods over a total period of 8 h (Post). 10 mM of the GABA-A-receptor (GABA-A-R) antagonist SR-95531 (gabazine) (Thermo Fisher Scientific) was injected into the yolk of fertilised one-cell stage wildtype zebrafish eggs as described elsewhere [53]. At 5 dpf the locomotor activity was tracked. Wildtype larvae were exposed to 0.1 mM of the GABA-B-receptor (GABA-B-R) antagonist CGP-55845 (Hello Bio) or to Danieau’s solution with 0.1% DMSO (control) for 48 h (3–5 dpf). At 5 dpf, both solutions were replaced with Danieau’s solution without any added substance and locomotion was recorded. 3 dpf old *foxp2*^+/+^ and *foxp2*^+/−^ siblings were bathed in 25 ml of 0.05 mM muscimol (Merck KGaA) diluted in Danieau’s solution or in Danieau’s solution only (control) until 5 dpf. During the incubation period, the solution was replaced daily. At 5 dpf, when locomotion was recorded, it was exchanged for Danieau’s solution without any added substance. An 8 mM stock solution of the psychostimulant methylphenidate (MPH, Merck KGaA) was further diluted to a working concentration of 0.024 mM in Danieau’s solution. 5 dpf old larvae were tracked in 1 ml Danieau’s solution for 10 min, comprising a 5 min habituation and a 5 min test phase (Pre). Afterwards, 500 µl Danieau’s solution was replaced by 500 µl of 0.024 mM MPH working solution (0.012 mM final concentration) or 500 µl Danieau’s solution only (control) [54]. After an incubation period of 1 h, the locomotor activity was recorded again by applying the same protocol (Post).

### Data analysis and statistics

Data analysis was performed in RStudio (RStudio 1.3.959, RStudio Team (2020). RStudio: Integrated Development for R. RStudio, PBC, Boston, MA). Data sets were tested for normal distribution by the Shapiro–Wilk’s test and for equality of variances by Levene’s test. Group differences were calculated distribution-dependent by unpaired *t*-test or unpaired Wilcoxon sign ranked test for two samples or by one-way ANOVA or Kruskal–Wallis rank-sum test for multiple samples. In case of multiple group comparison, we applied Tukey’s HSD post hoc test for parametric and Dunn’s post hoc test for non-parametric data. The false discovery rate was controlled by the Benjamini–Hochberg adjustment. The general significance level was defined as 0.05. To pool or compare behavioural data obtained by two versions of the applied tracking software, data frames were standardised using z-score transformation. Effect sizes were determined by Cliff’s delta in the *effsize* package (Torchiano, 2016; Effsize—a package for efficient effect size computation). Appropriate sample sizes were calculated using the software G*Power3.1.9.4 [55] with *α* and *β* equals 0.05.

## Results

### *foxp2* expression in the developing zebrafish CNS

We used RNA in situ hybridisation to visualise the spatio-temporal distribution of *foxp2* transcripts in zebrafish at embryonic and early larval stages (24–72 h post fertilisation (hpf)). Anatomical abbreviations are listed in Table S4. Expression of *foxp2* is first detectable in the telencephalon (Tel) at 24 hpf (Fig. 1A, Supplementary Fig. 1A–B) and maintained until 72 hpf (Fig. 1A, Supplementary Fig. 1C–J, K, M, O, Q). During subsequent developmental stages, this pattern is further accompanied by expression in the hypothalamus (H) and the ventral tegmentum (vTg) (Supplementary Fig. 1C, D, K) starting at 30 hpf, and in the posterior tuberculum (PT), the thalamus (Th), the preoptic region (Po), the optic tectum (TeO) and the medulla oblongata (MO) starting at 36 hpf (Fig. 1A, Supplementary Fig. 1E–J, O-R). At 72 hpf, *foxp2* transcripts are present in the inner nuclear layer (INL) and the ganglion cell layer (GCL) of the retina (Supplementary Fig. 1S) and dorsally along the spinal cord (SC) (Supplementary Fig. 1T).

### Generation of a *foxp2* loss-of-function mutation by CRISPR/Cas9

To perform functional studies of *foxp2* in zebrafish, we generated a *foxp2* mutant line with a CRISPR/Cas9 induced 40 bp deletion (Fig. 1B, Supplementary Fig. 2A). The frame shift-induced premature stop codon disrupts the amino acid sequence N-terminal of the zinc-finger domain (Fig. 1C). The nuclear localisation signals, the leucine-zipper, forkhead and C-terminal binding protein 1 binding domains are located downstream of the induced stop codon. Hence, we expect a complete loss of these domains of the Foxp2 protein. The human R328X truncation mutation, which is situated N-terminal to the zinc-finger domain, creates an unstable *FOXP2* gene product that remains in the cytoplasm and lacks DNA-binding and transactivation capacity [56]. Since our mutation and the R328X mutation cause a premature stop codon within the similar gene region we expect similar aberrations for any remaining *foxp2* gene product in our mutant.

By real-time quantitative PCR (qPCR) we detected a gene dose-dependent reduction of *foxp2* transcripts in mutants compared to wildtype siblings (Fig. 1D, Table S5), indicating nonsense-mediated decay. Simultaneously, we revealed an upregulation of *foxp1a*, while *foxp1b* expression was not significantly altered. Further, we found a significant upregulation of *cntnap2b*, a transcriptional target of Foxp2 [57], thus indicating a *foxp2* loss-of-function in our mutant.

Homozygous *Foxp2* knock-out mice are postnatal lethal, while heterozygous mutants exhibit a mild to moderate developmental delay [58]. Homozygous alleles for the here reported mutation in zebrafish caused alterations in the development or inflation of the swim bladder in about 20% of the cases (Fig. 1E). In heterozygous mutants, we observed no gross morphological malformations. We found no significant size differences for any of the evaluated parameters (Supplementary Fig. 2B) and no increased apoptosis in *foxp2*^+/^^−^ or in *foxp2*^−/−^ at 24 hpf (Supplementary Fig. 2C). However, genotyping of older stages revealed that unlike *foxp2*^+/−^, *foxp2*^−/−^ do not reach adulthood.

### Homozygous deletion of *foxp2* results in disorganised commissures and tracts in zebrafish larvae

Identification of Foxp2 targets involved in neurite development, together with altered neurite morphology following *Foxp2* mutations in animal and cell culture models, suggest a role for Foxp2 in neurite growth and guidance [18, 19, 22, 25, 28]. In zebrafish, the effect of Foxp2 on neuritogenesis is unclear. Earlier investigations reported no effect on axon pathfinding in a zinc-finger nuclease-induced *foxp2* mutant [59], while another zinc-finger nuclease-induced mutation affecting *cntnap2* caused delayed commissure formation in zebrafish larvae [60]. To investigate whether the here reported mutation of *foxp2* affects the organisation of main commissures and tracts in the developing brain, we performed anti-acetylated tubulin staining on mutant and wildtype siblings. At 20 and 24 hpf, we noted a disorganised appearance of the anterior commissure (AC), the post-optic commissure (POC) and the supra-optic tract (SOT) in *foxp2*^−^^*/−*^, while the situation in *foxp2*^*+/*^^−^ was unchanged (Supplementary Fig. 2D–E). We observed no qualitative effects on the commissure and tract structures at 28 hpf and 5 dpf, which suggests that homozygous *foxp2* mutant alleles induce a delay in the early formation of commissures and tracts, which recovers until 28 hpf.

### *foxp2* deletion increases locomotor activity in zebrafish larvae

**Fig. 2:**
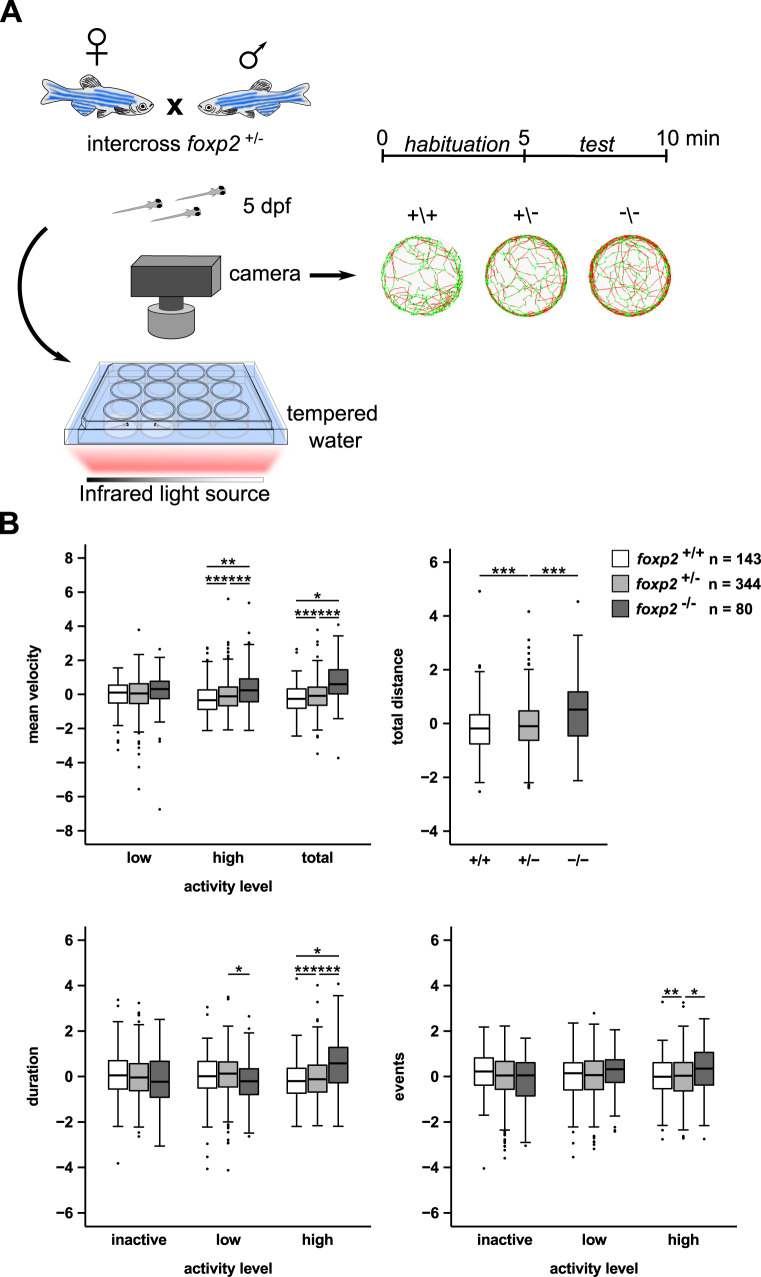
Increased locomotor activity in larval *foxp2* mutants. **A** Crossing scheme and behavioural setup applied for locomotor tracking in 5 dpf old *foxp2*^+/+^, *foxp2*^+/−^ and *foxp2*^−^^/−^. Circles illustrate representative swim tracks of individual fish with inactive (<0.2 cm/s, black), low activity (>0.2 cm/s and <1 cm/s, green) and high activity (>1 cm/s, red). **B** Locomotor activity of *foxp2*^+/+^ (white), *foxp2*^+/−^ (light grey) and *foxp2*^−/−^ (dark grey) analysed for mean velocity (top left) in low or high activity, or combined (total), total distance swum (top right), duration (bottom left) and events (bottom right) of inactivity, low and high activity. Raw data was standardised using z-score transformation. *P < 0.05, **P < 0.01, ***P < 0.001.

Foxp2 is implicated in motor functions in mammals as well as in flies. Besides complex motor tasks such as sequenced orofacial movements during speech in humans [61], motor skill learning, vocalisation or vocal imitation in mammals or birds [24, 26, 62, 63] findings in fruit flies have demonstrated a role for Foxp2 in basic motor functions such as locomotion [18]. To investigate whether Foxp2 plays a role in the regulation of basic motor functions in zebrafish larvae, we compared locomotion of 5 dpf old *foxp2* mutants and wildtype siblings (Fig. 2A). For both *foxp2*^+/−^ and *foxp2*^−/−^, we measured a significant increase in locomotor activity displayed as an increased swimming velocity and distance (Fig. 2B). In addition, the velocity and the duration of “high activity” were significantly increased for both mutant groups (Fig. 2B). Beyond that, *foxp2*^*+/−*^ mutants displayed a significantly elevated number of “high activity” swimming events (Fig. 2B).

### *foxp2* is expressed by GABAergic neurons

Previous studies performed to identify neuronal circuits underlying motor phenotypes in *Foxp2* mutants focused predominantly on dopamine-dependent neuromodulation in the striatum or homologous brain regions [22, 64]. In the striatum, *Foxp2* is expressed by GABAergic MSNs [28], which are modulated by dopamine and regulate motor functions [65]. Interestingly, *Foxp2* loss-of-function increases striatal direct pathway inhibition through reduced transcriptional repression of *Gad1* [29], which indicates a regulation of GABA-mediated inhibition as a functional role of Foxp2.

**Fig. 3:**
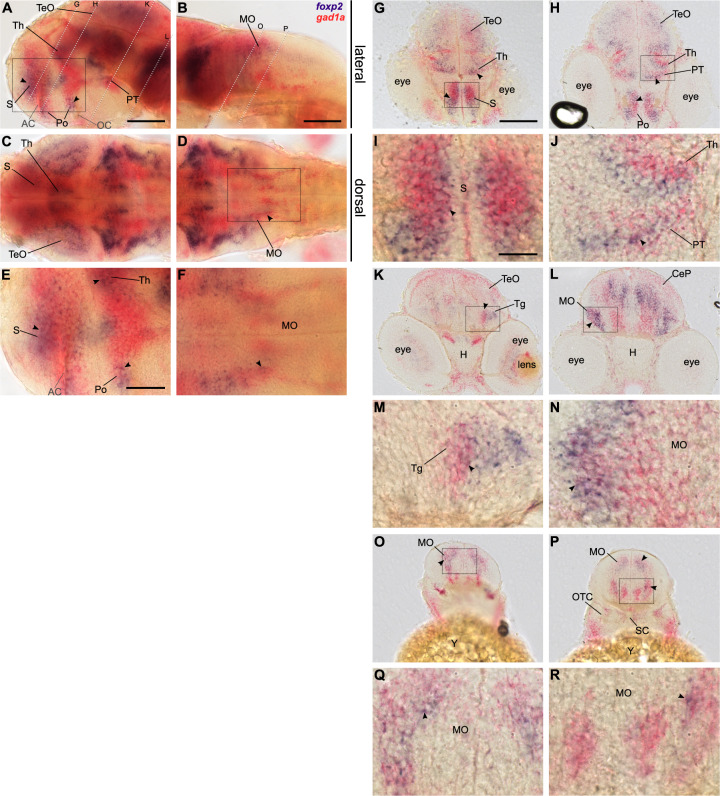
*foxp2* is expressed by a subset of *gad1a* positive neurons in the developing CNS. Double labelling of *foxp2* (blue) and *gad1a* (red) expression in 48 hpf old wildtype embryos using two-colour RNA ISH. Lateral (**A**, **B**, **E**) and dorsal (**C**, **D**, **F**) overview of embryonic CNS with anterior to the left. **E**, **F** Magnifications of boxed areas in **A** and **D**. Dashed lines indicate cutting sites for cross-sections displayed in **G**–**R**. **I**, **J**, **M**, **N**, **Q**, **R** magnifications of boxed areas in **G**, **H**, **K**, **L**, **O**, **P**, respectively. Arrows indicate sites of co-localisation. Abbreviations are listed in Table S4. Scale bars: 100 µm (overview), 50 µm (magnified images).

To investigate whether *foxp2* is expressed by GABAergic neurons of the developing zebrafish brain, particularly in regions involved in motor control, we performed two-colour RNA ISH for *foxp2* and *gad1a*. Since the expression pattern of both *GAD1* paralogs*, gad1a* and *gad1b*, are highly similar (Supplementary Fig. 3A–L), we decided for *gad1a* as GABAergic marker due to technical reasons. For all three stages examined, *foxp2* expression partially overlaps with *gad1a* expression in the subpallium (S), the preoptic region (Po), the thalamus (Th), the posterior tuberculum (PT) (Fig. 3A, C, E, Supplementary Figs. 4A, C, E and 5A, C, E) and the lateral and dorsal medulla oblongata (MO) (Fig. 3B, D, F, Supplementary Figs. 4B, D, F and 5B, D, F). Co-expression of *foxp2* and *gad1a* was further visualised and confirmed on cryo-sections in the aforementioned regions (Fig. 3G–R, Supplementary Figs. 4G-R and 5G–R). In addition, later stages display overlapping expression of *foxp2* and *gad1a* in the optic tectum (TeO) and the inner nuclear layer (INL) of the retina (Supplementary Fig. 5A, C, G, H, K). Hence, we confirmed that *foxp2* is expressed in a subset of GABAergic neurons in tel-, di-, mes- and rhombencephalon of the developing zebrafish brain with striking incidence for areas involved in motor functions.

### Interference with GABAergic signalling alters locomotor activity

The data described above confirm the presence of *foxp2* in GABAergic neurons in developing brain regions involved in motor control and show that *foxp2* loss-of-function results in increased locomotor activity. Interestingly, impaired Gad1 function is implicated in altered motor function [43, 66]. Hence, we hypothesised that Gad1-activity is important for the regulation of locomotor behaviour in zebrafish larvae. We applied two complementary strategies to interfere with Gad1 activity: a splice-inhibiting morpholino targeting the *gad1b* transcript or application of L-allylglycine, a direct Gad antagonist. Targeting *gad1b* instead of *gad1a* was motivated by a higher amino acid similarity compared to human GAD1 (Gad1a, 81%; Gad1b, 84%; ensembl.org, GRCz11).

**Fig. 4:**
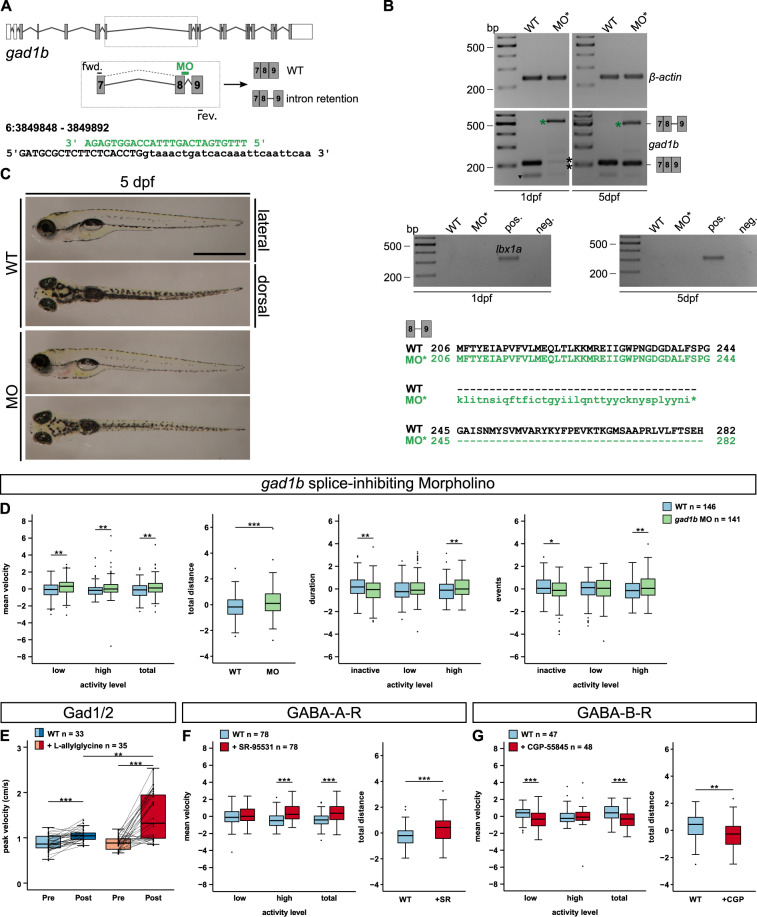
Effects of altered GABAergic signalling on locomotor activity in 5 dpf old zebrafish larvae. **A** *gad1b* exon–intron structure with coding (grey) and non-coding exons (white). *gad1b* splice-inhibiting morpholino (MO, green) and corresponding primer binding sites are indicated. Binding of the *gad1b*-MO causes retention of intron 8. **B**, top and centre The reduction of the *gad1b* wildtype transcript at 1 dpf (black asterisks, 208 bp) and the presence of the misspliced transcript at 1 and 5 dpf (green asterisk, 500 bp) was verified by RT-PCR. A primer off-target (black triangle) was detected below the wildtype *gad1b* transcript. Control PCRs on *beta-actin* (above, 239 bp) and *lbx1a* (below, 353 bp) confirmed comparable cDNA levels and absent genomic DNA contamination for all samples. **B**, bottom The predicted amino acid sequence of the misspliced *gad1b* transcript suggests a premature termination of translation in *gad1b* intron 8 (asterisk). **C** Overviews of a 5 dpf old *gad1b* morphant (MO) and an uninjected control larva (WT). Scale bar, 1 mm. **D** Locomotor activity of 5 dpf old *gad1b* morphant larvae (MO, green) compared to uninjected controls (WT, light blue). Locomotor activity was assessed by mean velocity in low or high activity, or combined (total), total distance swum, duration or events of inactivity, low and high activity. **E** Maximum mean velocity (of 5 min) reached within 8 h of tracking (peak velocity in cm/s) after incubation (Post) in 100 mM Gad-inhibitor L-allylglycine (dark red) or Danieau’s solution (control, dark blue). Maximum mean velocity (of 5 min) of the same individuals before incubation in L-allylglycine (Pre, light red) or Danieau’s solution (Pre, light blue). Line plot displays activity changes of individual larvae. **F** Locomotor activity of 5 dpf old wildtype larvae injected with 10 mM GABA-A-receptor antagonist SR-95531 (red) or water (light blue) at one-cell stage. **G** Effect of 0.1 mM GABA-B-receptor antagonist CGP-55845 on locomotor activity of 5 dpf old wildtype larvae (red) in comparison to Danieau’s solution incubated controls (light blue). **D**, **F**, **G** Raw data was standardised using z-score transformation. *P < 0.05, **P < 0.01, ***P < 0.001.

The *gad1b* splice-inhibiting morpholino caused retention of intron 8 detected at 1 and 5 dpf and a decreased amount of wildtype transcript at 1 dpf that partly recovered until 5 dpf (Fig. 4A–B, Supplementary Fig. 6A). The intron retention induces a frameshift and a subsequent premature stop codon. No gross morphological alterations were observed (Fig. 4C). Size measurements revealed no significant effect on head size or yolk diameter, but a slight reduction in body length (Supplementary Fig. 6B). Unlike *foxp2* mutants, *gad1b* morphants exhibited increased cell death (Supplementary Fig. 6C). However, wildtypes treated with L-allylglycine at 5 dpf showed no apparent changes in size or cell apoptosis (not shown), which may be attributed to the acute treatment strategy later in development.

During locomotor tracking, we found that *gad1b* knock-down induces a hyperactive phenotype similar to that of *foxp2* mutants (Fig. 4D). Further, we pharmacologically phenocopied this effect in wildtype individuals, however with a stronger effect and in absence of any developmental delay or anatomical alterations through the acute application of the Gad antagonist L-allylglycine (Fig. 4E). Taken together, these results show that transcriptional loss of *gad1b* or pharmacological blockage of Gad induces hyperactivity, a behavioural phenotype that resembles a genetic loss of *foxp2*.

We complemented our investigations by inhibiting either GABA-A-receptors (GABA-A-R) or GABA-B-receptors (GABA-B-R) and subsequent locomotor tracking. Interestingly, we measured opposite effects with increased locomotor activity in wildtype individuals treated with the GABA-A-R antagonist SR-95531 (Fig. 4F) and decreased locomotor activity upon exposure to the GABA-B-R antagonist CGP-55845 (Fig. 4G).

To summarise, by targeting different levels of GABAergic signalling, including the *gad1b* transcript, the Gad enzymatic activity and two receptors (GABA-A-R and GABA-B-R), we showed that GABAergic signalling plays an important role in the regulation of locomotor behaviour in zebrafish larvae.

### Increased locomotor activity in *foxp2* mutants is rescued by increased GABA-A-R-mediated inhibition

**Fig. 5:**
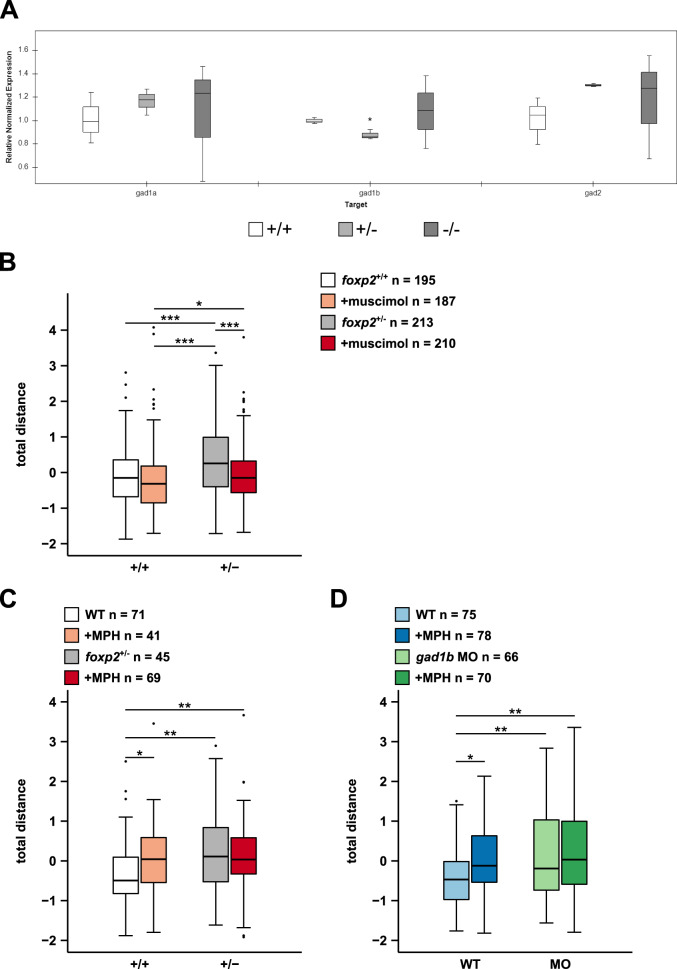
Muscimol and methylphenidate differentially affects locomotor activity in *foxp2*^*+/+*^ and *foxp2*^*+/−*^. **A** Transcript levels of *gad1a*, *gad1b* and *gad2* in *foxp2*^+/+^ (white), *foxp2*^+/−^ (light grey) and *foxp2*^−/−^ (dark grey) siblings assessed by qPCR. *P < 0.05. **B** Locomotor activity, displayed as total distance swum, of 5 dpf old *foxp2*^+/+^ and *foxp2*^+/−^ following a bath-application in 0.05 mM GABA-A-R agonist muscimol (light red and dark red, respectively) or Danieau’s solution (white and grey, respectively). **C** Locomotor activity, displayed as total distance swum, of 5 dpf old *foxp2*^+/+^ and *foxp2*^+/−^ after exposure to Danieau’s solution (white and grey, respectively) or 0.012 mM methylphenidate (MPH, light red and dark red, respectively). **D** Locomotor activity, displayed as total distance swum, of 5 dpf old *gad1b* morphants (MO) and wildtype controls (WT) exposed to Danieau’s solution (light green and light blue, respectively) or 0.012 mM MPH (dark green and dark blue, respectively). Raw data was standardised using z-score transformation. *P < 0.05, **P < 0.01, ***P < 0.001.

Motivated by our qPCR results, showing a significant reduction in the amount of *gad1b* transcript in *foxp2*^+/−^ and a tendency to higher *gad1a* and *gad2* transcript levels in *foxp2*^+/−^ and *foxp2*^−/−^ (Fig. 5A), we hypothesised that the observed increase in locomotor activity of *foxp2* mutants might be caused by an alteration of Gad*-*regulated GABAergic inhibition. Due to the significant downregulation of *gad1b* expression and the consistent phenotype across *foxp2* knock-out and *gad1b* knock-down, we suggest a reduction of GABAergic inhibition as one possible cause.

To compensate for a potential reduction of GABAergic inhibition in *foxp2*^+/−^ we applied the GABA-A-R agonist muscimol. Under control conditions, we confirmed the previously observed increase in locomotor activity of *foxp2*^+/−^ compared to *foxp2*^+/+^ (Fig. 5B). However, in response to muscimol, *foxp2*^+/−^ significantly reduced their locomotor activity to a level similar to that of untreated *foxp2*^+/+^, whereas the locomotor activity of *foxp2*^+/+^ was not significantly altered compared to the control conditions (Fig. 5B). Hence, we showed that the increased locomotor activity of *foxp2*^+/−^ can be rescued by increasing GABA-A-R-mediated inhibition and that *foxp2*^+/−^ and *foxp2*^+/+^ respond differently to muscimol.

### MPH treatment differentially alters locomotor activity in *foxp2* mutants and *gad1b* morphants in comparison to wildtype controls

Both *FOXP2* and *GAD1* are identified as potential ADHD risk genes [1, 39]. Since hyperactivity represents one of the core behavioural endophenotypes in ADHD symptomatology, we tested whether we could rescue the observed hyperactivity of *foxp2*^+/−^ and *gad1b* morphants by exposure to methylphenidate (MPH), a well-established psychostimulant in ADHD pharmacotherapy [46].

Interestingly, the locomotor hyperactivity of *foxp2*^+/−^ was not affected by MPH treatment (Fig. 5C), whereas in *foxp2*^+/+^ MPH increased locomotor activity. We made similar observations in *gad1b* morphants and wildtype controls. While MPH induces increased locomotor activity in wildtype controls (Fig. 5D), it does not significantly alter locomotion of *gad1b* morphants (Fig. 5D). Thus, wildtypes, *foxp2*^*+/−*^ and *gad1b* morphants exhibit a differential response to MPH, suggesting that direct or indirect MPH targets are altered upon *foxp2* and *gad1b* loss-of-function.

## Discussion

Here we explore the hypothesis that Foxp2 and GABAergic signalling are part of a biological network involved in the regulation of behavioural activity. We show that *foxp2* loss-of-function results in increased locomotor activity and demonstrate that *foxp2* is expressed by GABAergic neurons in several brain regions involved in motor functions. Intriguingly, disruption of Gad1 or GABA-A receptor activity causes hyperactivity, resembling the phenotype observed in our *foxp2* mutants. By application of the GABA-A-R agonist muscimol, we are able to rescue the hyperactive phenotype induced by the *foxp2* loss-of-function. Together these findings support the hypothesis that *foxp2* regulates locomotor activity via GABAergic signalling. This provides one possible mechanism by which behavioural phenotypes, such as altered activity, seen in NDDs may be explained.

By qPCR, we note a significant gene dose-dependent reduction of *foxp2* transcripts and a concurrent upregulation of *foxp1* transcripts in mutants. Compensatory upregulation of paralogous genes is a well-known phenomenon under knock-out conditions and may account for a milder or even a lack of an expected phenotype [67], especially since Foxp1 and Foxp2 share several transcriptional targets [68]. In addition, recent expression data suggests that Foxp1 and Foxp2 may function as direct regulatory opponents in some neurons [69], whereas providing coordinated regulation in others [68]. Hence the upregulation of *foxp1a* (and *foxp1b*), whether due to genetic compensation or de-repression, can cause phenotypic buffering, but may as well be a crucial mechanism in the observed *foxp2* mutant phenotype. Since we observed a gene dose-dependent phenotype in expression, morphology and behaviour we conclude, that the upregulation of *foxp2* paralogs cannot (fully) compensate for the loss of *foxp2*. However, we cannot exclude that the phenotypes would be more severe or different in double or triple mutants where additional *foxp* homologs are disrupted. Besides *foxp1a*, we found a significant upregulation of *cntnap2b* and *mef2cb* (Supplementary Fig. 7, Table S5), thereby confirming well-known targets of Foxp2 [19, 57] also in the zebrafish. Our results indicate that Foxp2 acts as a transcriptional repressor on *cntnap2b*. A previously published zinc-finger nuclease-induced *foxp2* zebrafish mutant rather suggest transcriptional activation of *cntnap2b* [59]. These seemingly conflicting findings may be explained by a context-dependent function of Foxp2, such as different genetic backgrounds or stage-dependent interaction with distinct transcriptional co-factors. Observations from other model systems show that Foxp2 usually acts as a repressor, but can also activate gene expression [4, 5]. Finally, many of the potential Foxp2 targets tested by qPCR in the present study (Supplementary Fig. 7, Table S5) and which are previously identified NDDs risk genes [1, 3, 54, 70, 71] exhibit increased expression levels, although only a few of them reach statistical significance. This supports the suggestion that Foxp2 is a central actor in a molecular network affected in multiple NDDs.

The aforementioned zinc-finger nuclease-induced *foxp2* zebrafish mutant exhibited a normal morphological appearance [59]. The mutant was not examined for any behavioural phenotypes. In our study, we similarly conclude that the macroscopic morphology is normal, except for a loss of the swim bladder in about 20% of the *foxp2*^−^^*/−*^. However, our analysis of major neurite bundles in the brain revealed a transient disorganised appearance at early stages. This suggests a rapid recovery in neurite growth and a narrow critical window when *foxp2* influences major neurite growth. Notably, *cntnap2a* and *2b* mutants display a similar phenotype that recovers with progressing development [60]. Hence, altered expression of *cntnap2b* (and *cntnap2a*) might be responsible for the transiently disorganised neurite bundle structure in the here presented *foxp2*^*−/−*^ mutants. Our current analysis is not detailed enough to allow us to conclude about the fine organisation of neurites and synapses, but is an interesting path for future investigations, in particular with respect to *foxp2*-positive populations of GABAergic neurons.

The spatio-temporal expression analysis of *foxp2* confirms previous findings [7] and reveals a conform picture of *foxp2* transcript distribution in teleost fish [10, 13] and a comparable distribution of *FOXP2/Foxp2* for mammals, amphibians, reptiles and birds [6, 8, 9, 11, 12, 14–17]. Thus, our findings support the notion of an evolutionary conserved expression of *FOXP2/Foxp2/foxp2* in the vertebrate brain. Of particular interest in our *foxp2* expression analysis is the high resemblance to the distribution of GABAergic neuron populations [72]. By performing a double RNA ISH, we show that *foxp2* and *gad1a* overlap and co-localise especially in distinct areas essential for motor functions [65]. These observations are of major interest with regard to motor deficits observed in various Foxp2 mutant lines [58, 62] and propose altered GABAergic signalling as a crucial factor for further investigations on motor impairments in *foxp2/Foxp2* deficient animals and *FOXP2*-associated human disorders.

Aberrations in GABAergic signalling are implicated in NDDs. Accordingly, a familial duplication of a cluster of four GABA_A_ receptor subunit genes segregates with multiple NDDs [38], polymorphisms in *GAD1* are associated in particular with the hyperactive/impulsive domain in children with ADHD [39], variants of the GABA-transporter gene *GAT1 (SLC6A1)* correlates with the risk for ADHD in a case–control study [40] and multiple genetic variants with known direct relation to GABAergic or glutamatergic signalling are associated to symptom severity in ADHD [41]. Further, brain imaging consistently shows alterations in brain regions with substantial GABAergic neuron populations in ADHD, e.g. prefrontal cortex, basal ganglia and cerebellum [46]. Magnetic resonance spectroscopy revealed altered levels of GABA in the prefrontal, anterior cingulate, motor and primary somatosensory cortices as well as in the basal ganglia in ADHD patients [47–49]. In a study of monozygotic twins discordant for ADHD, brain imaging combined with epigenetic evaluations demonstrated alterations in striatum and cerebellum correlating with differential methylation of GABAergic genes [50]. Similarly, animal models support the notion of GABA playing a yet underrated role in the pathophysiology of NDDs. Genetic mouse models with impaired function GABAergic signalling components mirror core ADHD symptoms such as hyperactivity, impaired sustained attention and increased impulsivity [42–45]. Interestingly, hyperactivity in the *Gat1* knock-out mice was rescued by stimulant application [45]. Further, *Cdh13* knock-out mice display ADHD-like phenotypes and Cdh13 localises to GABAergic neurons [34, 73]. Both mouse *Cntnap2* and zebrafish *cntnap2a/2b* loss-of-function exhibit loss of GABAergic neurons and display hyperactivity [60, 74].

The observations described above, combined with the co-localisation of *gad1a* and *foxp2* in regions involved in motor control, raise the question whether FOXP2/Foxp2 may be part of a biological network, which regulates GABAergic signalling and thereby motor functions affected in NDDs. In the present study we address this question and provide two major findings. Firstly, interference with *gad1b*, Gad or GABA-A-Rs results in an increased locomotor activity similar to what we find after *foxp2* impairment, thus we demonstrate that GABAergic signalling influences locomotor activity in zebrafish. Secondly, the hyperactive phenotype of *foxp2* mutants is rescued by muscimol-induced GABA-A-R activation. This reveals that the hyperactivity induced by *foxp2* impairment is, at least partly, caused by a deficit in GABAergic signalling that can be compensated for by a direct activation of GABA-A-Rs. The opposite effects on locomotor activity upon application of GABA-A-R or GABA-B-R antagonists can be explained by the disparate synaptic localisation and downstream functions of the two receptor classes.

It is well established that pharmacological substances used to treat ADHD such as methylphenidate (MPH) primarily target catecholaminergic neurotransmission [46]. However, effects of MPH on GABAergic signalling have been noted both in humans and in animal models [75–77]. In addition, substances with known GABAergic effects may be beneficial in treatment of ADHD symptoms [33]. We therefore tested the impact of MPH on our *foxp2* and *gad1b* loss-of-function situations. As expected, we found that the activity level of wildtypes is increased in response to MPH. Similar responses are reported in multiple experimental situations and are attributed to the stimulant effect of MPH [78]. In contrast, neither *foxp2*^*+/−*^ nor *gad1b* morphants responded to MPH. This suggests an altered function of direct or indirect MPH target(s) upon *foxp2* or *gad1b* loss-of-functions. Interestingly, activation of D_1_ or D_2_ receptors increases or decreases movement initiation, respectively, in zebrafish larvae [79]. Notably the D_1_ receptor encoding gene is a proposed transcriptional target of Foxp2 [64, 80] and both are expressed by GABAergic forebrain neurons, which exhibit altered function upon *Foxp2* loss-of-function [29]. Hence, we speculate that the reduced MPH sensitivity of *foxp2* mutants and *gad1b* morphants may be caused by reduced D_1_ receptor levels in *foxp2* mutants and/or altered downstream signalling capacity through impaired GABA synthesis in both *foxp2* mutants and *gad1b* morphants.

In summary, we conclude that Foxp2 regulates GABAergic signalling and that this is a crucial mechanism for the regulation of locomotor activity in zebrafish larvae. Further, we propose that inhibition of downstream targets is mediated by GABA-A-R activation. Currently we can only speculate about the identity of the involved neuronal circuitries. Previous investigations in zebrafish showed co-expression of *foxp2* and a *dlx5* and *dlx6* reporter transgene marking striatal basal ganglia [7]. Interestingly, Dlx genes are essential for the differentiation of GABAergic forebrain neurons [81], and here we noted co-localisation of *foxp2* and *gad1a* in the ventral forebrain. Together with previously reported alterations in striatal GABAergic inhibition as well as structural and functional striatal aberrations upon *foxp2* loss-of-function [19, 25, 28, 29], our observations indicate that the effect may be rooted in the GABAergic neurons of the ventral forebrain. In conclusion, we provide experimental support for the hypothesis that FOXP2 is a likely player in the development of NDDs and that dysregulation of the GABAergic component impacting on the E/I balance should obtain increased attention in future research agendas addressing pathomechanism of NDDs.

## Supplementary information

Supplementary Materials and Methods

Supplementary legends

Supplementary Figure 1

Supplementary Figure 2

Supplementary Figure 3

Supplementary Figure 4

Supplementary Figure 5

Supplementary Figure 6

Supplementary Figure 7

Supplementary Table 1

Supplementary Table 2

Supplementary Table 3

Supplementary Table 4

Supplementary Table 5
