# Supplementary material for: Increased locomotor activity via regulation of GABAergic signalling in foxp2 mutant zebrafish—implications for neurodevelopmental disorders
Source: Transl Psychiatry. 2021 Oct 14;11:529. doi: 10.1038/s41398-021-01651-w (PMC8517032; doi:10.1038/s41398-021-01651-w)
Supplement: Supplementary file 1 — Supplementary Materials and Methods [file 41398_2021_1651_MOESM1_ESM.docx]

**Supplementary Materials and Methods**

**Whole-mount RNA** ***in situ*** **hybridisation**

To generate a cDNA template for *foxp2* RNA ISH probe synthesis, a target site was amplified by PCR (for primers, see Table S1). The PCR product was cleaned by the GenElute PCR Clean-Up Kit (Merck KGaA), cloned into pCRII with the TA Cloning Kit Dual Promoter (Thermo Fisher Scientific) and verified by Sanger sequencing. The plasmid was linearised (*Not*I, Thermo Fisher Scientific) and purified (GenElute PCR Clean-Up Kit, Merck KGaA) before the RNA probe was *in vitro* transcribed with SP6 RNA polymerase and DIG RNA Labeling Mix (Merck KGaA). The final RNA probe was purified by LiCl and ethanol precipitation.

Whole-mount RNA ISH was performed in accordance with Thisse and Thisse, 2008 (1) and is described in detail in (2). In brief, specimens fixed in 4% PFA overnight at 4°C were washed in PBS containing 0.1% tween-20 (PBST) and dehydrated through a methanol (MeOH) series with final storage in 100% MeOH at -20°C. Upon ISH, the samples were rehydrated into PBST and permeabilised by Proteinase K treatment (10 µg/ml). After post-fixation in 4% PFA for 20 min at room temperature (RT) and extensive washes in PBST, the specimens were incubated in hybridisation buffer containing 5 mg/ml torula yeast RNA type VI (Merck KGaA) for 1 h at 65°C. Afterwards, the specimens were transferred into hybridisation buffer containing the RNA ISH probe (dilution 1:100) and incubated overnight at 65°C. On the second day, the specimens were treated through stringency washes at 65°C in decreasing concentrations of hybridisation buffer diluted with 2x saline-sodium citrate (SSC) buffer and finally washed for 1 h at 65°C in 0.05x SSC. Subsequently, the samples were washed in PBST and incubated in ISH blocking buffer (PBST with 2% normal sheep serum (NSS) and 2 mg/ml bovine serum albumin (BSA)) for 1 h at RT. For anti-digoxigenin (anti-DIG) immunolabelling, the specimens were incubated (2h at RT) in sheep anti-digoxigenin Fab fragments conjugated with Alkaline Phosphatase (AP; anti-DIG-AP, Merck KGaA) diluted 1:5000 in ISH blocking buffer. After additional washing steps in PBST at RT and overnight at 4°C, specimens were washed in alkaline tris-buffer (pH 9.5) at RT. The AP activity was revealed by nitroblue tetrazolium/5-bromo-4-chloro-3-indolylphosphate (NBT/BCIP) (Merck KGaA) in alkaline tris buffer. The enzymatic reaction was stopped by washes in PBST at RT and overnight at 4°C, followed by a fixation step in 4% PFA for 20 min at RT. Finally, specimens were stored in 80% glycerol in PBST in the dark.

*Two-colour in situ hybridisation*: For two-colour RNA ISH, *gad1a* (previously *gad67a*) cloned into pBluescript II (KS+) (kindly provided by Laure Bally-Cuif) was linearised (*EcoR*I, Thermo Fisher Scientific), purified (GenElute PCR Clean-Up Kit, Merck KGaA) and transcribed using T3 RNA polymerase and fluorescein (FLUO) RNA Labeling Mix (Merck KGaA). For hybridisation, both digoxigenin- and fluorescein-labelled probes were mixed and diluted (both 1:100) in hybridisation buffer. For immunolabelling, the embryos were first incubated in sheep anti-fluorescein-AP Fab fragments (anti-FLUO-AP) diluted 1:2000 in ISH blocking buffer. The embryos were then washed in tris-buffer (pH 8.2) and the AP activity was revealed by the application of SIGMAFAST fast red TR/naphthol AS-MX phosphate (4-chloro-2-methylbenzenediazonium/3-hydroxy-2-naphthoic acid 2,4-dimethylanilide phosphate) tablets dissolved in 0.1M Trizma buffer (Merck KGaA). The enzymatic reaction was stopped by washes in PBST and the anti-fluorescein-AP Fab fragments were detached by an incubation step in PBST at 68°C for 2h. Then, blocking, anti-DIG-AP immunolabelling and detection of the AP activity was performed as described above.

**Cryosections**

Cryosections were prepared as describe elsewhere (3). In brief, specimens stored in 80% glycerol were washed and cryoprotected in PBS with 15% sucrose overnight at 4°C. Next, the specimens were embedded in 7.5% gelatine dissolved in PBS with 15% sucrose. Blocks were cut with a scalpel and snap-frozen in 2-methylbutane pre-cooled with liquid nitrogen. Frozen cryoblocks were cut transversally with a section thickness of 20 µm on a cryostat (Microm HM 500 OM). Sections were collected on SuperFrostPlus slides (Thermo Fisher Scientific) mounted with 80% glycerol and a cover slip. The sections were stored in the dark at 4°C until imaging.

**Immunohistochemistry**

Immunohistochemical stainings were performed on whole-mount 20-28 hpf embryos with the yolk removed or on 5 dpf old embryos where the eyes, the yolk, the jaw, and the skin overlying the brain were removed using a pair of forceps after fixation. The genotype of immuno-stained *foxp2* mutants was determined prior to storage and image acquisition. Therefore, the tail of each embryo was cut and used for gDNA extraction and genotyping PCR as described above.

*Anti-Acetylated Tubulin (AcTub):* Embryos were fixed in 4% PFA for 1 h per 24h of development at RT and washed in PBS. Subsequently, the specimens were dehydrated through increasing concentrations of MeOH in PBS with 0.5% triton X-100 (0.5% PBT) and stored in 100% MeOH for at least one night at -20°C. Before antibody application, the specimens were rehydrated through a reverse MeOH series into 0.5% PBT. Afterwards, they were digested with Proteinase K (20-28 hpf: 10 µg/ml; 5 dpf: 40 µg/ml) at RT. After a quick wash in 0.5% PBT, the specimens were fixed in 4% PFA for 20 min at RT. Following washes in 0.5% PBT, the specimens were blocked in blocking buffer (PBT with 1% DMSO, 10% NSS and 2 mg/ml BSA) for 1 h at RT. They were then incubated overnight at 4°C in monoclonal mouse anti-acetylated tubulin primary antibody (IgG2b, #T7451, Merck KGaA, RRID:AB_609894) diluted 1:500 in blocking buffer. After washes in 0.5% PBT at RT, the specimens were incubated overnight at 4°C in secondary goat anti-mouse IgG (H+L) conjugated to Alexa Fluor 488 (A-11029, Thermo Fisher Scientific, RRID:AB_138404) diluted 1:1000 in blocking buffer. Afterwards, the specimens were washed in 0.5% PBT at RT and stored in 80% glycerol in PBST at 4°C in the dark.

*Anti-Cleaved Caspase 3 (cCasp3)*: 24 hpf old embryos were fixed in 4% PFA for 3 h at RT and dehydrated through increasing concentrations of MeOH in PBS with 0.8% triton X-100 (0.8% PBT) and finally stored in 100% MeOH. The specimens were permeabilised in 100% acetone for 7 min at -20°C. Following rehydration in 50% MeOH in 0.8% PBT for 1 h at -20°C, the specimens were washed in ddH_2_O and 0.8% PBT at RT. Subsequently, the specimens were blocked for 1 h at RT in blocking buffer (0.8% PBT with 1% DMSO, 10% NSS and 2 mg/ml BSA), before incubation for three days at 4°C with a polyclonal rabbit anti-Cleaved Caspase-3 (Asp175) primary antibody (#9661, Cell Signalling Technology, RRID:AB_2341188) diluted 1:500 in blocking buffer. After extensive washes in 0.8% PBT, the embryos were incubated for two days at 4°C in secondary goat anti-rabbit IgG (H+L) conjugated to Alexa Fluor 488 (A-11034, Thermo Fisher Scientific, RRID:AB_2576217) diluted 1:1000 in blocking buffer. Finally, the specimens were washed in 0.8% PBT and stored in 80% glycerol in PBST at 4°C.

**Image acquisition and processing**

Image acquisition of RNA ISH labelling was performed with a Zeiss Axiophot light microscope equipped with a Zeiss AxioCam MRc digital camera and the AxioVision Rel.4.8 Ink. software. Images were taken with three different Plan-Neofluar objectives (2.5x/0.075, Ph2 20x/0.50 and 40x/0.75, Carl Zeiss AG). Sizes were measured on images taken with a Leica M205 FA fluorescence microscope equipped with a Leica DFC 420C digital camera and the Leica Application Suite V3.8 Ink. software. Images were recorded through a Leica Planapo 1.0x objective (Leica Camera AG). Sizes of fixed embryos (24 hpf) were measured on images using the ImageJ image processing package Fiji 2.0.0 (4). For head size measurements, the head area was traced using the midbrain-hindbrain boundary as posterior boundary. The yolk diameter was measured between the most ventral centre of the eye and the edge between circular and elongated yolk sac. General length was measured between the dorsal part of the midbrain-hindbrain boundary and the most posterior end of the tail, excluding the fin. Size measurements are all specified in squared pixels (pixel^2^). Images on anti-AcTub and anti-cCasp3 stainings were taken at a Zeiss LSM 780 confocal microscope equipped with a Lasos Argon 488nm laser and the ZEN 2012 SP1 software. Images were taken with a Plan APO 20x/0.8 objective. Brightness of confocal images was adjusted in Fiji 2.0.0. Images on the anti-AcTub stainings were processed by a background subtraction based on the sliding paraboloid algorithm of ImageJ (Sternberg et al. 1983) followed by a convoluted background subtraction with a Gaussian convolution filter in the ImageJ toolbox BioVoxxel (Brocher 2015). Quantification of commissure and tract formation was done on processed confocal images from 24 hpf *foxp2^-/-^*, *foxp2^+/-^* and *foxp2^+/+^* siblings by five fully blinded raters, based on a qualitative rating scale. The rating scale defines four levels of commissure and tract formation established based on images from anti-AcTub stainings in *foxp2*^+/+^. (0) commissures/tracts are entirely absent, (1) single commissure/tract fibres visible, (2) several commissure/tract fibres visible, but reduced, (3) commissures/tracts are present, but reduced terminal distribution (4) wildtype-like commissure and tract formation. Image arrangements into final figures were done using the vector graphics software Inkscape 1.0.1. (www.inkscape.org).

**Generation and genotyping of a *foxp2* exon 10 CRISPR/Cas9 mutant line**

The CRISPR/Cas9 target site was selected based on the algorithm CHOPCHOP (5). Two oligonucleotide templates for the synthesis of the single guide RNA (sgRNA) (Table S1) were annealed and ligated into the *Bsa*I sites (*Eco31*I/*Bsa*I, Thermo Fisher Scientific) of the pDR274 vector (kindly provided to the public by the Keith Joung Lab and Addgene #42250) by T4 DNA ligase (Thermo Fisher Scientific). Positive clones were determined by colony PCR (Oligo2 as forward and M13 uni (-21) as reverse primer, Table S1) and confirmed by Sanger sequencing. The plasmid containing the annealed oligonucleotides was linearised (*Dra*I, Thermo Fisher Scientific) and used for *in vitro* transcription by a custom-made T7 RNA polymerase (kindly provided by Thomas Ziegenhals and Utz Fischer). Subsequently, the generated sgRNA was purified by Roti-phenol/chloroform/isoamylalcohol (Carl Roth GmbH & Co. KG) RNA isolation.

Generated sgRNAs were injected (190 ng/µl) together with the Cas9-NLS protein (300 ng/µl, *S. pyogenes*, New England Biolabs) into the animal pole of fertilised one-cell stage zebrafish eggs. gDNA of injected embryos was extracted and used as template for PCR to confirm induced indel mutations at the expected target site (*foxp2* primers, Table S1). The generated PCR products were separated on a 3% high-resolution NuSieve 3:1 agarose gel (Lonza Group) in 1x tris-borate-EDTA buffer, and Sanger sequenced for confirmation.

Injected F_0_ embryos were raised and tested for germline transmission of indel mutations by outcrossing with AB/AB wildtypes and genotyping the offspring. Positive F_0_ fish were then outcrossed again with AB/AB wildtypes to breed the F_1_ generation. Individual *foxp2* F_1_ adult mutants were identified to analyse the type of induced mutations. Therefore, the PCR product generated from the F_1_ fish was purified (GenElute PCR Clean-Up Kit, Merck KGaA) and TA cloned into the pCRII vector (TA Cloning Kit Dual Promoter, Thermo Fischer Scientific). Extracted plasmid from positive clones was sent for Sanger sequencing (LGC Genomics) to exactly characterise the indel mutations. Finally, the deletion mutation described in Fig. 1 was selected. For final mutation verification, gDNA of *foxp2^-/-^* F_3_ mutants was PCR amplified and Sanger sequenced (LGC Genomics). For the experiments described below, F_2_ or F_3_ embryos and larvae generated by outcrosses of *foxp2^+/-^* to AB/AB or intercrosses of *foxp2^+/-^* were used.

**Injection of splice-inhibiting morpholino and verification via RT-PCR**

A *gad1b* splice-inhibiting morpholino oligonucleotide (GeneTools, Table S1) was diluted in nuclease-free water (Thermo Fisher Scientific) and injected into the animal pole of fertilised one-cell stage AB/AB zebrafish eggs (0.5 nl of 0.5 mM morpholino). Injected eggs were raised until 1 and 5 days post fertilisation (dpf). Total RNA from five morphant (MO) and five uninjected control embryos (WT) from each developmental stage was extracted with phenol-chloroform followed by LiCl and ethanol precipitation and treated with DNase I (Merck KGaA). Subsequently, the RNA samples were tested for genomic DNA (gDNA) contamination by PCR (*lbx1a* primers, Table S1) and then transcribed into cDNA using an oligo-dT primer and the RevertAid First Strand cDNA Synthesis Kit (Thermo Fisher Scientific). Successful reverse transcription was confirmed by a beta-actin control PCR (*actb1* primers, Table S1) and morpholino-derived splicing defects were verified by PCR for *gad1b* (*gad1b* primers, Table S1) and confirmed by Sanger sequencing.

**RNA isolation and quantitative real-time PCR (qPCR)**

Quantitative real-time RT-PCR (qPCR) was performed on 5 dpf old *foxp2^-/-^*, *foxp2^+/-^* and *foxp2^+/+^* siblings. The tail of each embryo was cut for gDNA extraction and subsequent genotyping. Remaining tissue was stored in RNAprotect Tissue Reagent (Qiagen N.V.) for RNA extraction. Total RNA was isolated from 10 pooled embryos for each genotype using the RNeasy^®^ Mini Kit (Qiagen N.V.). The samples were lysed in lysis buffer with β-mercaptoethanol (10 µl/ml) and homogenised using the TissueLyser II (Qiagen N.V.) before total RNA was extracted according to manufacturer’s instructions. The isolated RNA was treated with DNase I (Thermo Fisher Scientific) and the final RNA concentration was determined spectrophotometrically. For cDNA synthesis the SuperScript^TM^ IV Reverse Transcriptase Kit (Thermo Fisher Scientific) was used. Each target gene (Table S2) was represented by a triplet (technical replicates) of each biological sample (n=3) for each genotype (*foxp2*^+/+^, *foxp2*^+/-^ and *foxp2*^-/-^). As negative controls, no RT control (NRT) and no template control (NTC) were included. Each reaction contained a mixture of SYBR^TM^ Select Mastermix for CFX (Thermo Fisher Scientific), the respective cDNA (diluted 1:20) and the corresponding primer pair. The annealing temperature was set to 60°C. qPCR was run on a CFX384 Touch Real-Time PCR Detection System (Bio-Rad Laboratories, Inc.). Final quantification and calculation were conducted with the CFX Maestro software (Bio-Rad Laboratories, Inc.) and the comparative C_t_ (2-ΔΔCt) method. Significant group differences were determined by applying a one-way ANOVA with the significance level set to 0.05.

**Supplementary References**

1. Thisse C, Thisse B. High-resolution in situ hybridization to whole-mount zebrafish embryos. *Nat Protocols* 2008; **3**: 59-69.

2. Lechermeier CG, Zimmer F, Lüffe TM, Lesch K-P, Romanos M, Lillesaar C, et al. Transcript Analysis of Zebrafish GLUT3 Genes, slc2a3a and slc2a3b, Define Overlapping as Well as Distinct Expression Domains in the Zebrafish (Danio rerio) Central Nervous System. *Front Mol Neurosci* 2019; **12**: 199.

3. Reuter I, Jäckels J, Kneitz S, Kuper J, Lesch K-P, Lillesaar C. Fgf3 is crucial for the generation of monoaminergic cerebrospinal fluid contacting cells in zebrafish. *Biology Open* 2019; **8**: bio040683.

4. Schindelin J, Arganda-Carreras I, Frise E, Kaynig V, Longair M, Pietzsch T, et al. Fiji: an open-source platform for biological-image analysis. *Nature Methods* 2012; **9**: 676-82.

5. Labun K, Montague TG, Gagnon JA, Thyme SB, Valen E. CHOPCHOP v2: a web tool for the next generation of CRISPR genome engineering. *Nucleic Acids Res* 2016; **44**: W272-6.
