# Supplementary material for: Increased locomotor activity via regulation of GABAergic signalling in foxp2 mutant zebrafish—implications for neurodevelopmental disorders
Source: Transl Psychiatry. 2021 Oct 14;11:529. doi: 10.1038/s41398-021-01651-w (PMC8517032; doi:10.1038/s41398-021-01651-w)
Supplement: Supplementary file 2 — Supplementary legends [file 41398_2021_1651_MOESM2_ESM.docx]

**Supplementary Figure legends**

**Figure S1. *foxp2* expression pattern in the developing zebrafish labelled by whole-mount RNA *in situ* hybridisation.** (Left panel) Lateral (A, C, E, G, I) and dorsal (B, D, F, H, J) overview of *foxp2* expression pattern in 24, 30, 36, 48 and 72 hpf old wildtype zebrafish. (Right panel) Magnifications of boxed areas in C, D and I, J displayed from lateral (K, L, O, P) and dorsal (M, N, Q, R) views. Magnifications of boxed areas for remaining stages (24, 36 and 48 hpf) are displayed in Fig. 1. S shows the eye from a dorsal view. T shows the spinal cord from a lateral view. Arrow indicates staining in the spinal cord. All images are oriented with anterior to the left. Abbreviations are listed in Table S4**.** The developmental trajectory of *foxp2* expression in the developing zebrafish is described in the main text in more detail. Scale bar, 200 µm (overview) and 100 µm (magnifications).

**Figure S2. Genetic and anatomical descriptions of *foxp2* mutants.** (A) DNA sequencing traces for genotyping PCR product derived from *foxp2*^+/+^, *foxp2*^+/-^ and *foxp2*^-/-^. *foxp2*^-/-^ traces miss 40 nt (between red and blue box) confirming the deletion mutation presented in Fig. 1B. *foxp*^+/-^ show multiple traces from the deletion mutation onwards (end of the blue box) representing a mixture of wildtype and mutated allele. (B) Size measurements (given in squared pixel (pixel^2^)) of head area (top), yolk diameter (center) and total body length (bottom) in 24 hpf old *foxp2*^+/+^ (white, n=10), *foxp2*^+/-^ (light grey, n=24) and *foxp2*^-/-^ (dark grey, n=19). Neither *foxp2*^+/-^ nor *foxp2*^-/-^ showed significant size differences in head, yolk, or total length in comparison to *foxp2*^+/+^. (C) Anti-cleaved Caspase 3 staining (cCasp3) revealed no general increase in apoptosis in the central nervous system (CNS) of 24 hpf old *foxp2*^+/-^ and *foxp2*^-/-^ compared to *foxp2*^+/+^. The right column displays magnifications of boxed areas in the left column. Arrows indicate examples of apoptotic (cCasp3-positive) cells. Scale bar, 100 µm. (D) Evaluation of the average commissure and tract formation for the anterior commissure (AC), the post-optic commissure (POC) and the supra-optic tract (SOT). Quantification was performed by five fully blinded raters, based on a qualitative rating scale ranging from no commissure/tract formation (0) to full commissure formation (4, for details, see material and methods). Evaluation was performed on confocal images depicted from 24 hpf old *foxp2*^+/+^ (n=3), *foxp2*^+/-^ (n=4) and *foxp2*^-/-^ (n=6) embryos immunohistochemically stained for anti-acetylated tubulin (AcTub). *foxp2*^-/-^ show a significant alteration of the commissure and tract formation for the AC, POC and SOT at 24 hpf. *P<0.05, **P<0.01, ***P<0.001. (E) Comparison of anti-AcTub staining (green) across four developmental stages in *foxp2*^+/+^, *foxp2*^+/-^ and *foxp2*^-/-^. Alteration of commissure and tract formation is observable for AC, POC and SOT in *foxp2*^-/-^ at 20 hpf and 24 hpf. These alterations were no longer observable at 28 hpf and 5 dpf. All images displayed are oriented with anterior to the left. Scale bars, 50 µm (20 hpf, 24 hpf) and 100 µm (28 hpf, 5 dpf).

**Figure S3. *gad1a* and *gad1b* expression in 36 hpf old wildtype embryos.** Whole- mount RNA *in situ* hybridisation of *gad1a* (A-F) and *gad1b* (G-L) displayed from lateral (A-C and G-I) and dorsal views (D-F and J-L) with anterior to the left. Magnifications of boxed areas in overview images shown in A, D, G and J are displayed to the right. *gad1a* and *gad1b* show similar expression pattern with transcripts labelled in the telencephalon (Tel), thalamus (Th), hypothalamus (H), tegmentum (Tg) and medulla oblongata (MO). Scale bar, 200 µm (overview) and 100 µm (magnifications).

**Figure S4. Two-colour RNA *in situ* hybridisation for *foxp2* and *gad1a* in 36 hpf old wildtype embryos.** (A-F) Whole-mount preparations double labelled for *foxp2* (blue) and *gad1a* (red) displayed from lateral (A, B, E) and dorsal (C, D, F) views with anterior to the left. Magnifications of boxed areas in A, D are shown in E, F. Dashed lines indicate cutting sites for cross-sections (G-R). Boxed areas in G, H, K, L, O and P indicate magnified areas in I, J, M, N, Q and R, respectively. Arrows indicate regions where expressions of *foxp2* and *gad1a* overlap. For a detailed description of the expression pattern, see the main text. Abbreviations are listed in Table S4. Scale bar, 100 µm (overview) and 50 µm (magnifications).

**Figure S5. Two-colour RNA *in situ* hybridisation for *foxp2* and *gad1a* in 72 hpf old wildtype embryos.** (A-F) Whole-mount preparations double labelled for *foxp2* (blue) and *gad1a* (red) displayed from lateral (A, B, E) and dorsal (C, D, F) views with anterior to the left. Magnifications of boxed areas in A, D are shown in E, F. Dashed lines indicate cutting sites for cross-sections (G-R). Boxed areas in G, H, K, L, O and P indicate magnified areas in I, J, M, N, Q and R, respectively. Arrows indicate regions where expressions of *foxp2* and *gad1a* overlap. For a detailed description of the expression pattern, see the main text. Abbreviations are listed in Table S4. Scale bar, 100 µm (overview) and 50 µm (magnifications).

**Figure S6. Genetic and anatomical descriptions of *gad1b* splice-morphants.** (A) cDNA sequencing traces of the *gad1b* PCR product from wildtype (208 bp) and misspliced transcript (500 bp) from *gad1b* splice-morphants (MO). As expected, the wildtype transcript comprises exon 8 and exon 9, whereas the misspliced transcript spans exon 8, the entire intron 8 (green) and exon 9, and thus confirms the *gad1b*-MO-induced intron 8 retention. (B) Size measurements (given in squared pixels (pixel^2^)) of 24 hpf old *gad1b* morphant (MO, grey, n=10) and uninjected controls (WT, white, n=21) revealed no significant difference in head area (top left) and yolk diameter (top right). However, *gad1b* morphant showed a significant reduction in total body length (bottom) compared to wildtype controls. **P<0.01. (C) Anti-cleaved Caspase 3 (cCasp3) immunohistochemistry in 24 hpf old wildtype and morphant embryos revealed enhanced apoptosis in the CNS of *gad1b* morphants. Magnifications of boxed areas are displayed to the right, showing examples of cCasp3-positive (arrow heads) cells in a *gad1b* morphant. Images are displayed with anterior to the left. Scale bar, 100 µm (overview, left) and 20 µm (magnification, right).

**Figure S7. Relative expression analysis of potential Foxp2 target genes in *foxp2* mutants.** Expression of potential target genes is normalised against actin, beta 1 (*actb1*) and glyceraldehyde-3-phosphate dehydrogenase (*gapdh*). Expression analysis was performed for *foxp2*^+/+^ (white), *foxp2*^+/-^ (light grey) and *foxp2*^-/-^ (dark grey). Samples derived from *foxp2*^+/+^ are used as controls. For target gene list see Table S2. *P<0.05.

**Table S1. List of sequence, annealing temperature and related amplicon length/target site of oligonucleotides applied for RNA *in situ* hybridisation (RNA-ISH), splice-inhibiting morpholino-derived knock-down or gene editing by CRISPR/Cas9.** The protospacer adjacent motif (PAM) in the *foxp2* exon 10 target site is underlined. Amplicon length is given for genomic DNA (gDNA) and complementary DNA (cDNA) as template. Capital letters represent coding (exonic) and small letters non-coding (intronic) regions in the target site.

**Table S2. List of oligonucleotides applied for real-time quantitative PCR (qPCR), providing sequence, expected amplicon length and corresponding annealing temperature.**

**Table S3. List of pharmacological substances.**

**Table S4. List of anatomical abbreviations.**

**Table S5. Mean relative normalised expression, limit of upper and lower error bar and significance level for individual FoxP2 target genes assessed in *foxp2* mutants.** Raw data is normalised against the housekeeping genes actin, beta 1 (*actb1*) and glyceraldehyde-3-phosphate dehydrogenase (*gapdh*). Wildtype siblings (*foxp2^+/+^*) are used as controls.
