# Supplementary figures and images for: Increased locomotor activity via regulation of GABAergic signalling in foxp2 mutant zebrafish—implications for neurodevelopmental disorders
Source: Transl Psychiatry. 2021 Oct 14;11:529. doi: 10.1038/s41398-021-01651-w (PMC8517032; doi:10.1038/s41398-021-01651-w)

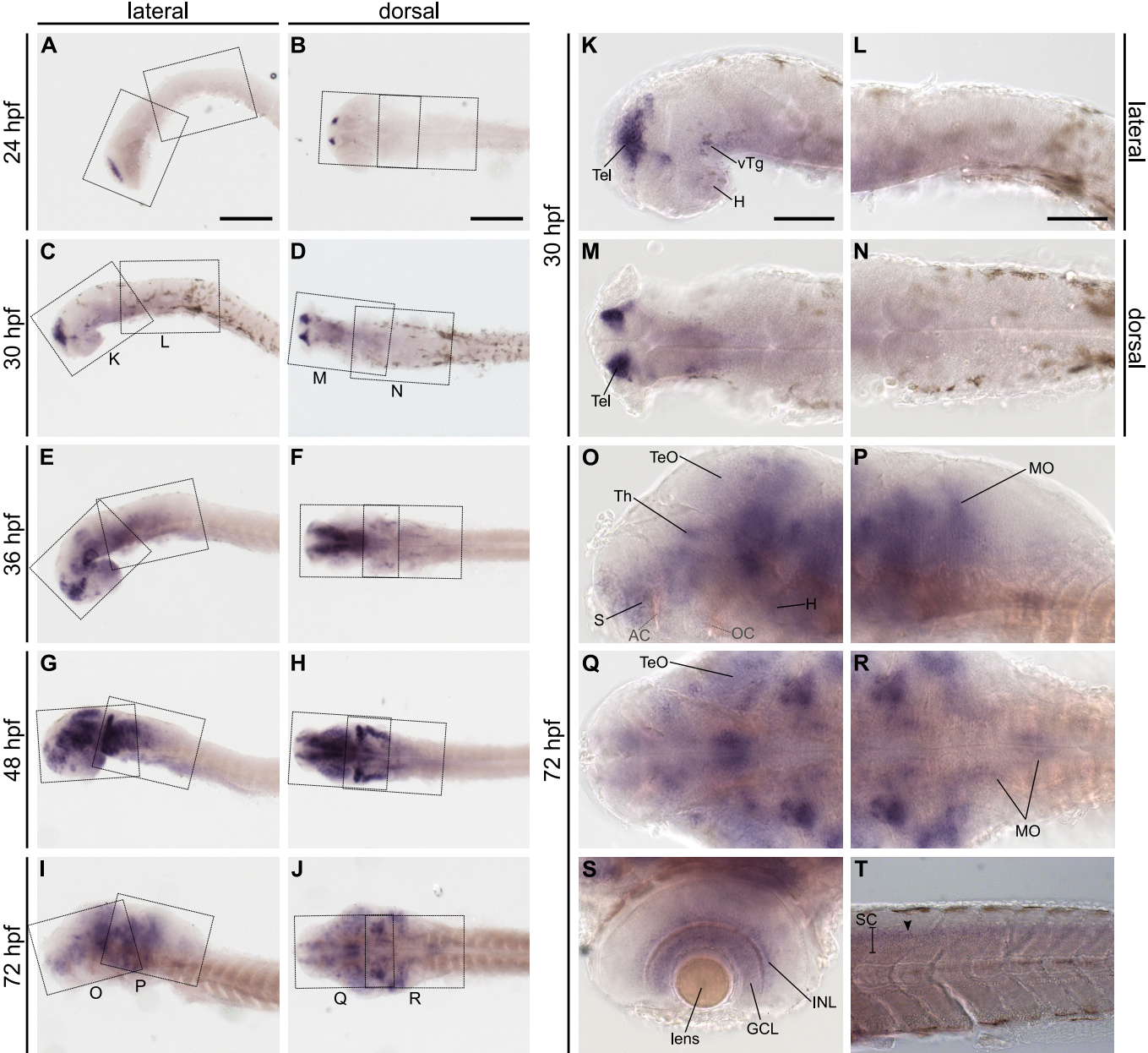

Supplement: Supplementary file 3 — Supplementary Figure 1 [file 41398_2021_1651_MOESM3_ESM.pdf]

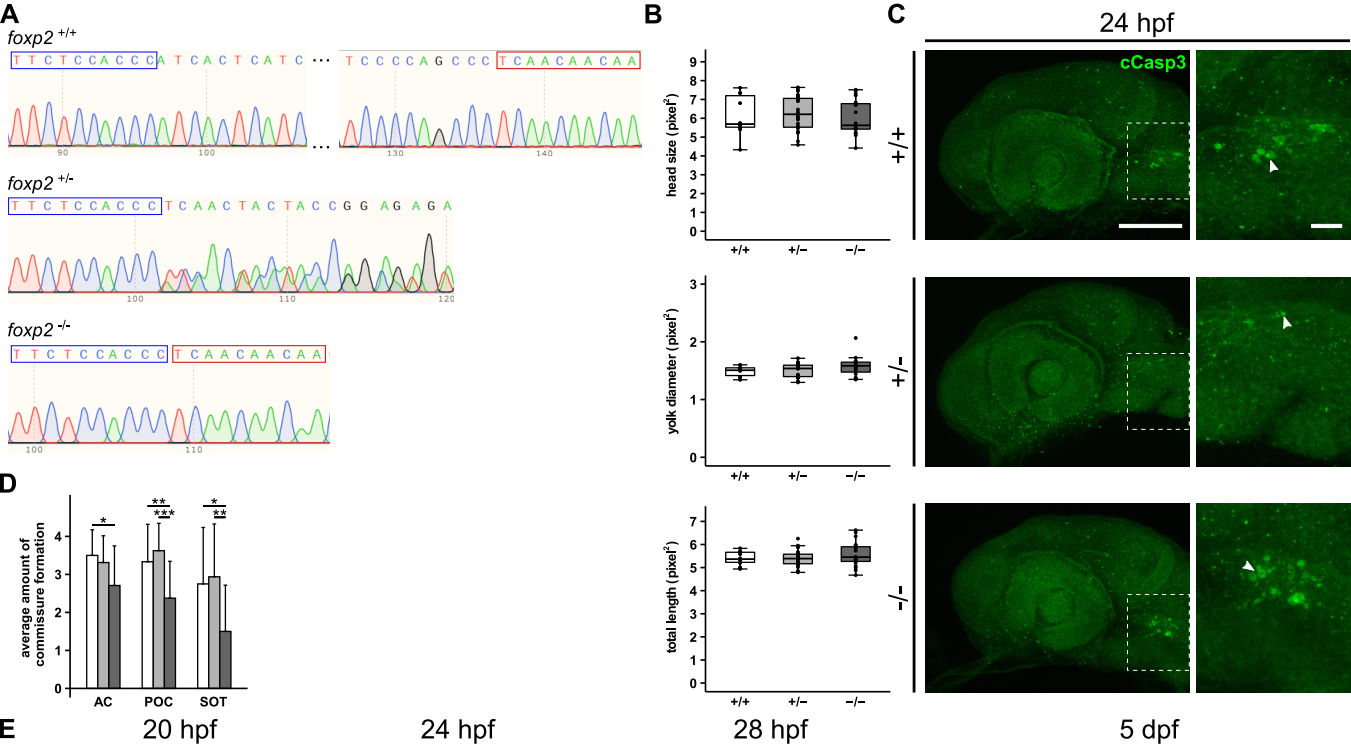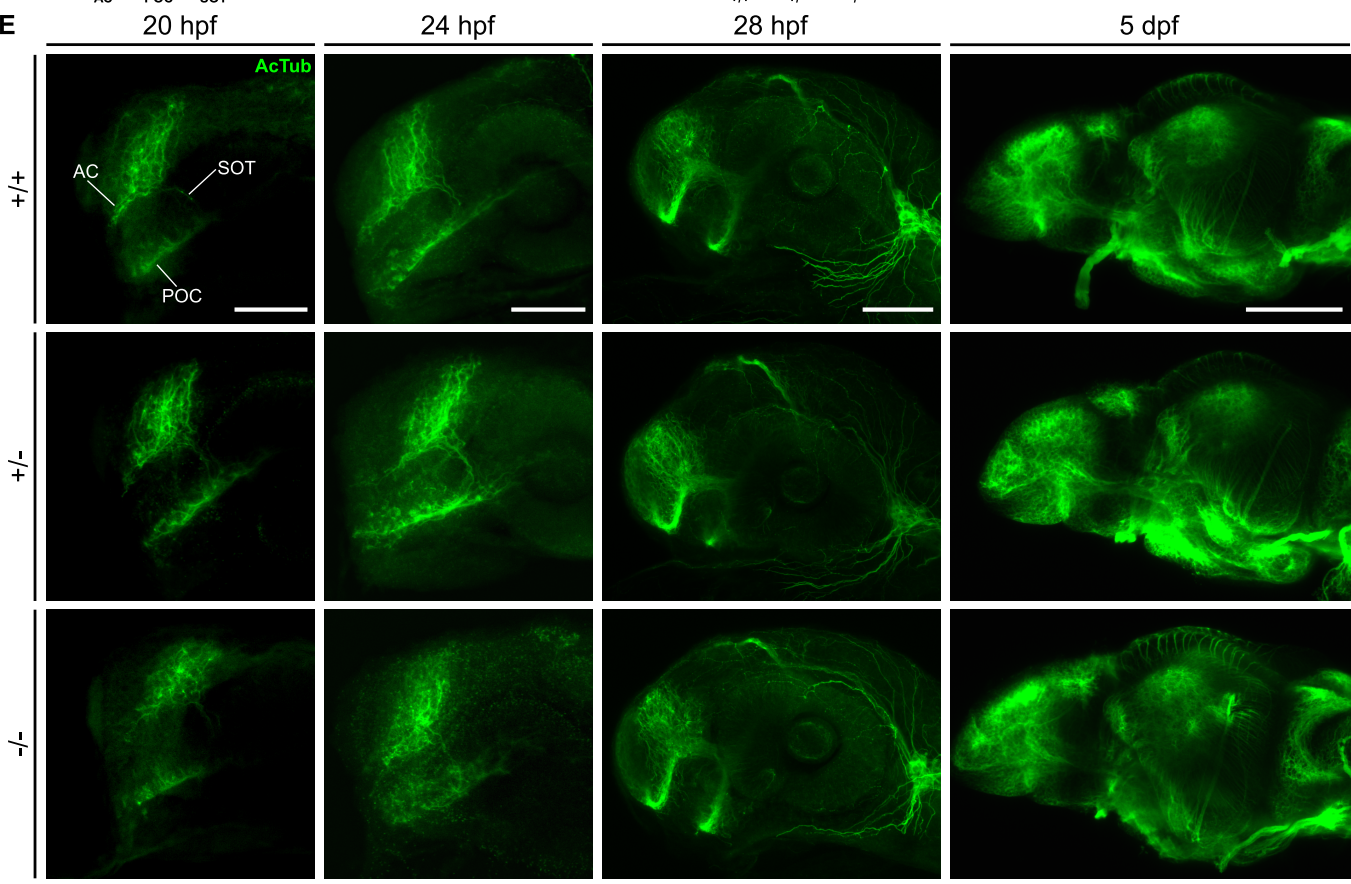

Supplement: Supplementary file 4 — Supplementary Figure 2 [file 41398_2021_1651_MOESM4_ESM.pdf]

*gad1a*

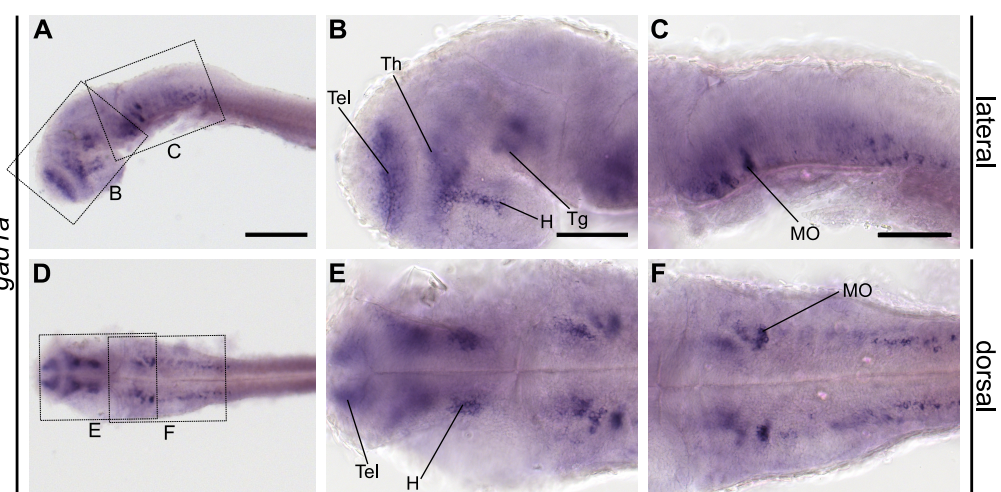

*gad1b*

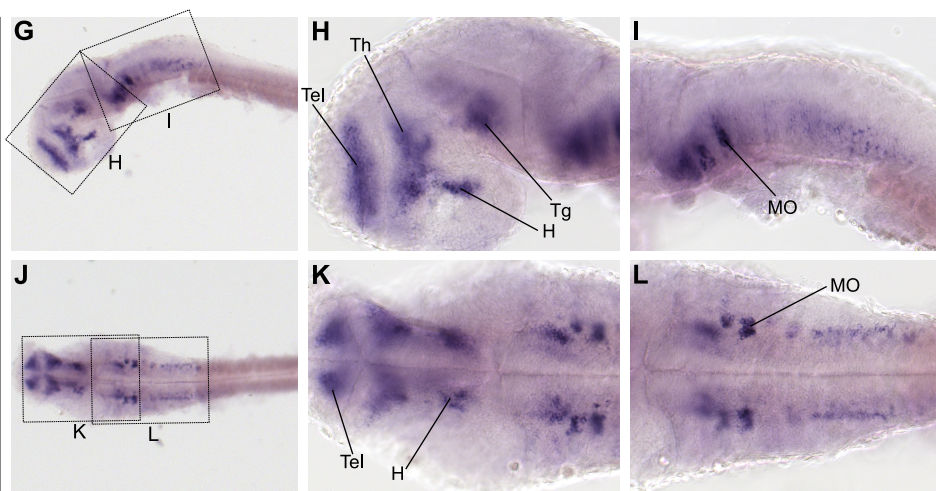

Supplement: Supplementary file 5 — Supplementary Figure 3 [file 41398_2021_1651_MOESM5_ESM.pdf]

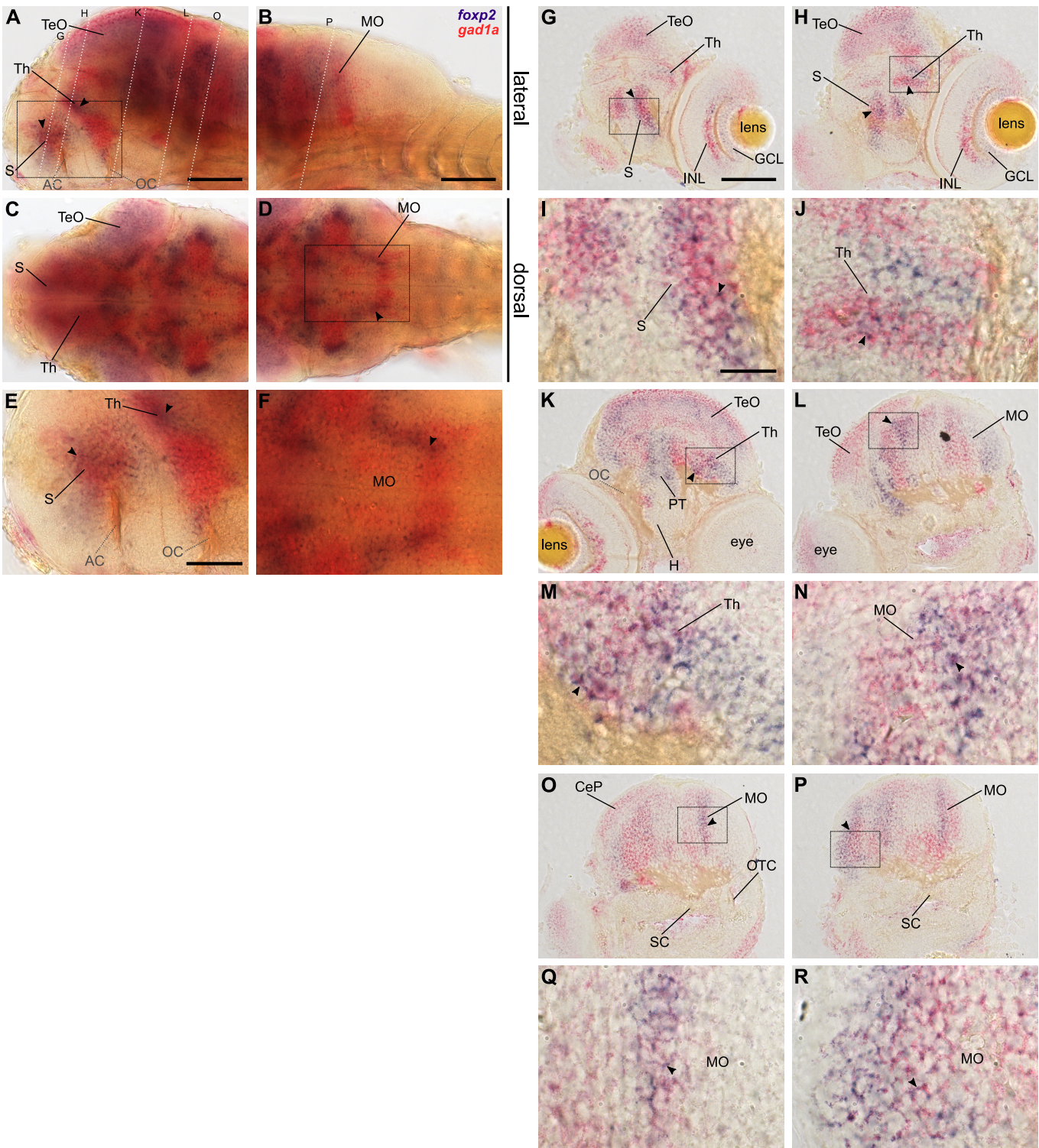

Supplement: Supplementary file 7 — Supplementary Figure 5 [file 41398_2021_1651_MOESM7_ESM.pdf]

**A***gad1b* MO 208-bp: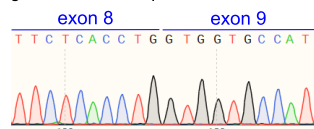*gad1b* MO 500-bp\* (intron 8 retention):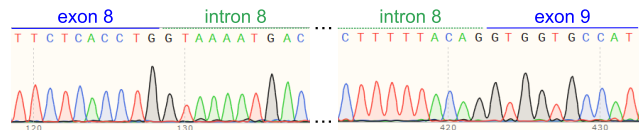**B**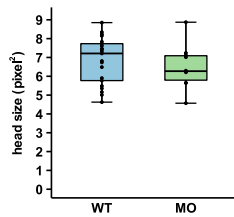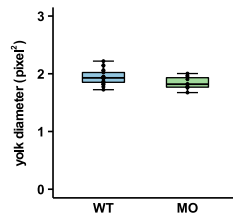**C**

24 hpf

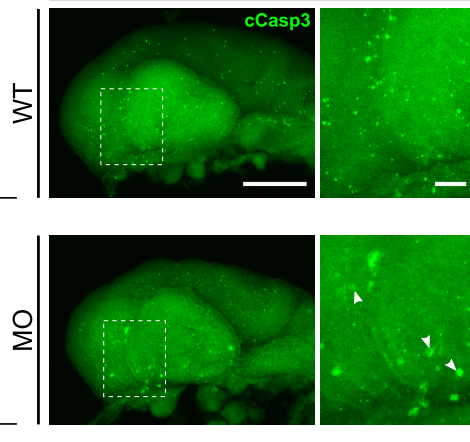

Supplement: Supplementary file 8 — Supplementary Figure 6 [file 41398_2021_1651_MOESM8_ESM.pdf]

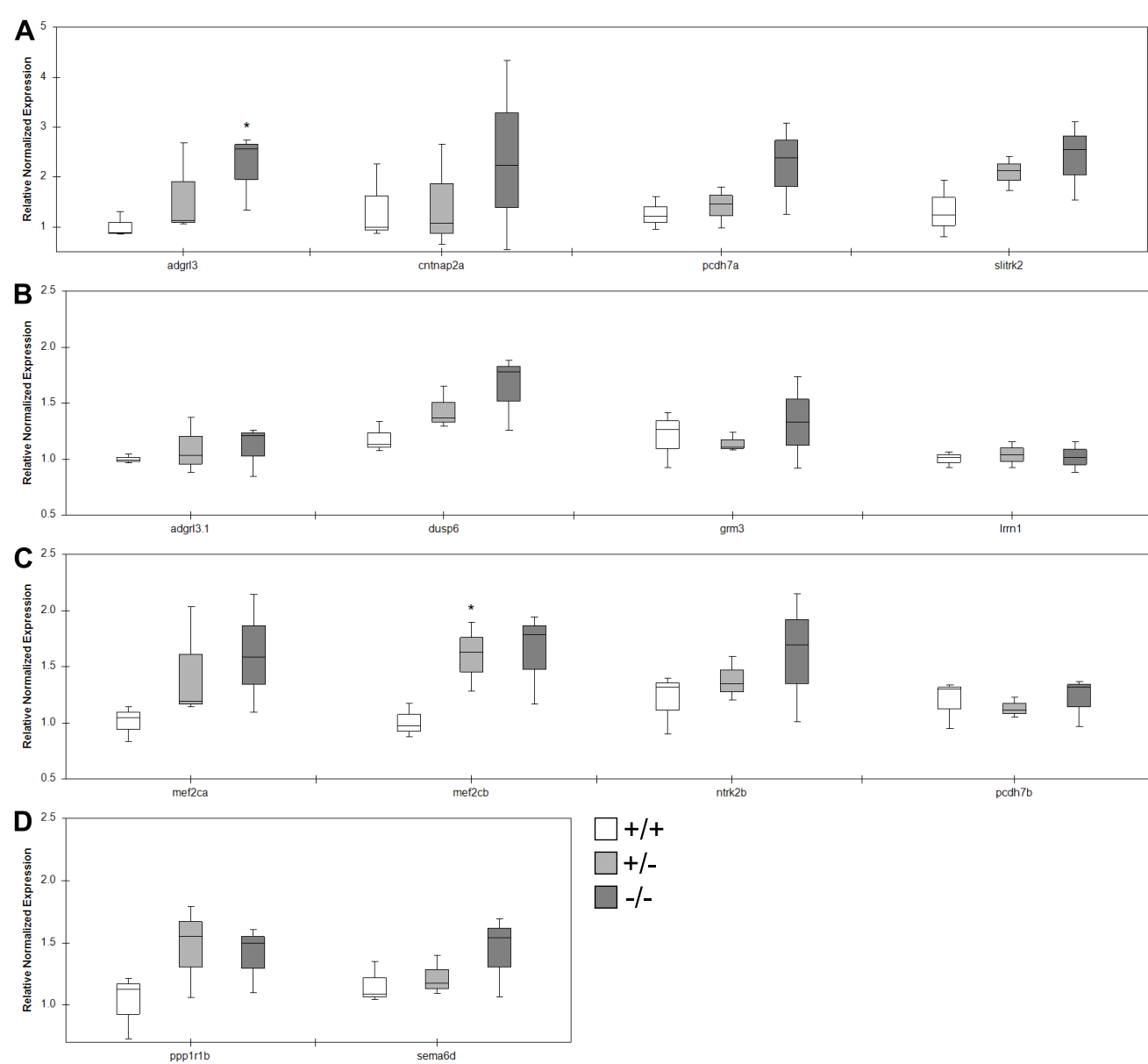

Supplement: Supplementary file 9 — Supplementary Figure 7 [file 41398_2021_1651_MOESM9_ESM.pdf]
