# Supplementary material for: Increased locomotor activity via regulation of GABAergic signalling in foxp2 mutant zebrafish—implications for neurodevelopmental disorders
Source: Transl Psychiatry. 2021 Oct 14;11:529. doi: 10.1038/s41398-021-01651-w (PMC8517032; doi:10.1038/s41398-021-01651-w)
Supplement: Supplementary file 10 — Supplementary Table 1 [file 41398_2021_1651_MOESM10_ESM.pdf]

Application	Oligo name	Gene symbol	Forward sequence (5'- 3')	Reverse sequence (5'- 3')	Amplicon length (gDNA)	Amplicon length (cDNA)	Ann. Temp. (°C)	Target site (5'- 3')
**RNA ISH**	*foxp2* exon 18 fwd./ 3'UTR rev.	*foxp2*	GGG TTA TGG GGC AGC TCT TA	CAC TTC AGT TCC GTG AGC CT	9839-bp	695-bp	59.5	
**Morpholino**	*gad1b* exon 8/ intron 8 Morpholino	*gad1b*		TTT GTG ATC AGT TTA CCA GGT GAG A				TCTCACCTGgtaaactgatcacaaa
	gad1b exon 7 fwd. / exon 9 rev.	*gad1b*	ATT GGT CTG GCT GGA GAA TG	ATT TAT ACC GCG CAA CCA TC	500-bp	208-bp	58	
	actb1 exon 2/3 fwd. / exon 3 rev.	*actb1*	CCC AGA CAT CAG GGA GTG AT	TCT CTG TTG GCT TTG GGA TT	0-bp	239-bp	53	
	lbx1a exon 1 fwd. / intron 1 rev.	*lbx1a*	TCC ACC TGC TAA CTC AAA CA	TTT AAC GAC CGT TTT CAC GA	353-bp	0-bp	56	
**CRISPR/Cas9**	*foxp2* exon 10 Oligo1 / exon 10 Oligo2	*foxp2*	TAG GAC ATG CTG TGA TGA GTG A	AAA CTC ACT CAT CAC AGC ATG TCC				GGACATGCTGTGATGAGTGATGG
	foxp2 exon 10 fwd. / exon 10 rev.	*foxp2*	CAA CTT TGG AAA GAC GTC ACT G	TGG GTA TGC GGT AGA ATA AAC C	268-bp	268-bp	58	
	M13 uni (-21) rev.			CAG GAA ACA GCT ATG AC				
