# Supplementary material for: Increased locomotor activity via regulation of GABAergic signalling in foxp2 mutant zebrafish—implications for neurodevelopmental disorders
Source: Transl Psychiatry. 2021 Oct 14;11:529. doi: 10.1038/s41398-021-01651-w (PMC8517032; doi:10.1038/s41398-021-01651-w)
Supplement: Supplementary file 11 — Supplementary Table 2 [file 41398_2021_1651_MOESM11_ESM.pdf]

Gene symbol	Gene name	Gene ID (Ensembl)	Forward primer sequence (5'- 3')	Reverse primer sequence (5'- 3')	Amplicon length (bp)	Ann. Temp. (°C)
actb1	actin, beta 1	ENSDARG00000037746	GCT GTT TTC CCC TCC ATT GTT	GGG CCT CAT CTC CCA CAT AG	91	62
adgrl3.1	adhesion G protein-coupled receptor L3.1	ENSDARG00000061121	ACT GGG AGA GTC TGC TGT GT	CCC GTG CGT TGT TGA GAA TG	117	62
adgrl3(.2)	adhesion G protein-coupled receptor L3(.2)	ENSDARG00000090624	GAC GAC GCC TTC CTC AAG AA	GCT CAG GAT CAG GCA GAG TG	80	62
cntnap2a	contactin associated protein 2a	ENSDARG00000058969	GCT GAT TTC CCC TTC AAT G	AGA TCA CCA CCG CAA TGA T	80	61
cntnap2b	contactin associated protein 2b	ENSDARG00000074558	AGG AGC GCA TCA GTC GAG AT	CGT GCA GAG GAT GGT GAA GAT	89	63
dusp6	dual specificity phosphatase 6	ENSDARG00000070914	TCT CGG AGG GCT CAG AAT CA	GGC TGC CAT CTG AGT CAG TT	92	62
foxp1a	forkhead box P1a	ENSDARG00000004843	GCT CTC GCC TCT GCC TAC	GTG TCT GGA CTC TGC TGC TC	114	62
foxp1b	forkhead box P1b	ENSDARG00000014181	AGA GGA AAT GAA TGG GGC CA	CTG GGT CCA TTG GCT CCT C	119	62
foxp2	forkhead box P2	ENSDARG00000005453	CCT GGA TAC TCC CCA CAC AC	ACA GTC CTC GTC CTC CAT GTT	78	61
gad1a	glutamate decarboxylase 1a	ENSDARG00000093411	CAG ATG GAG AGG AGA AAC GAC AT	ACC ATT GTT GTC CCG CAC T	85	66
gad1b	glutamate decarboxylase 1b	ENSDARG00000027419	ATG CCG AAC GGA GAC GAG	GCA CTC CAT CAT CAT TGC TTT G	75	63
gad2	glutamate decarboxylase 2	ENSDARG00000015537	GTG GAG AGG ATG AAG CGT CTG	GAC CAT GCG GAA GAA GTT GAC	123	62
gapdh	glyceraldehyde-3-phosphate dehydrogenase	ENSDARG00000043457	CGA TCA CTT TGT CAA GCT GGT	GCT GTA ACC GAA CTC ATT GTC A	89	62
grm3	glutamate receptor, metabotropic 3	ENSDARG00000031712	GAG CGG GCC AAG CAA CTA G	GAA CGT AGT GGG CAC TCA CA	116	63
lrrn1	leucine rich repeat neuronal 1	ENSDARG00000060115	TCC CGT TGC AAG GAA GGA TC	TCG TCT CGT TAC ATC CAC GC	84	62
mef2ca	myocyte enhancer factor 2ca	ENSDARG00000029764	TCA TCT GGG CTC CAT GAC C	GAC GGC AGA GAT AGG GCA GA	114	63
mef2cb	myocyte enhancer factor 2cb	ENSDARG00000009418	GCA GCA CTC TGC ACT CAG TC	CTT GAT GTG CAG GCT TTG AG	100	63
ntrk2b	neurotrophic tyrosine kinase, receptor, type 2b	ENSDARG00000098511	CTT CAC CTA TGG CAA GCA ACC	TAA CAC CCG ACC CTG TGT GAT	79	62
pcdh7a	protocadherin 7a	ENSDARG00000078898	TAG TGG GGT GGA GGA CTC AG	CTA CGC TCC CTT CTG GTG TG	116	64
pcdh7b	protocadherin 7b	ENSDARG00000060610	ACT AGG TGC CCT CCC TCT AC	CCA TAT GCT CTG CCT CAC CG	80	62
ppp1r1b	protein phosphatase 1, regulatory (inhibitor) subunit 1B	ENSDARG00000076280	TGG CTG AAG CTC AAA TGC AG	TTG TGC AGG ATG AGC AGA GC	105	62
sema6d	semaphorin 6D	ENSDARG00000002748	GTG ATG TCA AAT CAG CTG TGG A	AAA CGC TAG CAA CAC ACA CG	116	62
slitrk2	SLIT and NTRK-like family, member 2	ENSDARG00000006636	TCT ACG GGA CCC CCA GAA AA	CCA GCA CTT CGA GGT AGT CC	99	65
