# Supplementary material for: Increased locomotor activity via regulation of GABAergic signalling in foxp2 mutant zebrafish—implications for neurodevelopmental disorders
Source: Transl Psychiatry. 2021 Oct 14;11:529. doi: 10.1038/s41398-021-01651-w (PMC8517032; doi:10.1038/s41398-021-01651-w)
Supplement: Supplementary file 12 — Supplementary Table 3 [file 41398_2021_1651_MOESM12_ESM.pdf]

Substance	Target	Effect	Company	Applied on	Stock concentration	Diluted in	Concentrations tested (in Danieau's)	Treatment
L-allylglycine	Gad	antagonist	Santa Cruz Biotechnology	AB/AB	1000 mM	dH_2O	100 mM	8 h
gabazine hydrobromide / SR-95531	GABA-A-R	antagonist	Thermo Fisher Scientific	AB/AB		RNase-free water	10 mM	injection
muscimol hydrobromide	GABA-A-R	agonist	Merck KGaA	*foxp2* CRISPR	70 mM	dH_2O	0.05 mM	48 h
CGP55845 hydrochloride	GABA-B-R	antagonist	Hello Bio	AB/AB		Danieau's+ 0.1% DMSO	0.1 mM	48 h
methylphenidate hydrochloride	dopamine/ noradrenaline	reuptake inhibitor	Merck KGaA	*foxp2* CRISPR, *gad1b* MO	8 mM	dH_2O	0.012 mM	1 h
