# Supplementary material for: Increased locomotor activity via regulation of GABAergic signalling in foxp2 mutant zebrafish—implications for neurodevelopmental disorders
Source: Transl Psychiatry. 2021 Oct 14;11:529. doi: 10.1038/s41398-021-01651-w (PMC8517032; doi:10.1038/s41398-021-01651-w)
Supplement: Supplementary file 13 — Supplementary Table 4 [file 41398_2021_1651_MOESM13_ESM.pdf]

Abbreviation	Anatomical structure
AC	anterior commissure
ATN	anterior tuberal nucleus
CC	cerebellar crest
CCe	cerebellar corpus
CeP	cerebellar plate
CIL	central nucleus of the inferior lobe
Cpost	posterior commissure
DIL	diffuse nucleus of the inferior lobe
DT	dorsal thalamus
DTN	dorsal tegmental nucleus
EG	granular eminence
EN	entopeduncular nucleus
GCL	ganglion cell layer
H	hypothalamus
Ha	habenula
Hc	caudal zone of the periventricular hypothalamus
Hd	dorsal zone of periventricular hypothalamus
Hv	ventral zone of periventricular hypothalamus
INL	inner nuclear layer
IO	inferior olive
LCa	caudal lobe of cerebellum
LH	lateral hypothalamic nucleus
LLF	lateral longitudinal fascicle
LRL	lower rhombic lip
LVII	facial lobe
LX	vagal lobe
MFN	medial funicular nucleus
MLF	medial longitudinal fascicle
MO	medulla oblongata
MON	medial octavolateralis nucleus
NLV	lateral valvular nucleus
NIII/IV	oculomotor/trochlear nucleus
NXm	vagal motor nucleus
OB	olfactory bulb
OC	optic commissure
OT	optic tract
OTC	otic capsule
P	pallium
Pc	pretectal complex
PGZ	periventricular gray zone of optic tectum
Po	preoptic region
POC	post-optic commissure
PP	periventricular pretectal nucleus
PT	posterior tuberculum
PTN	posterior tuberal nucleus
PVO	paraventricular organ
RF	reticular formation
Rv	rhombencephalic ventricle
S	subpallium
SC	spinal cord
SOT	supra-optic tract
Tel	telencephalon
TeO	optic tectum
TeV	tectal ventricle
Tg	tegmentum
Th	thalamus
TL	longitudinal torus
TLa	lateral torus
TPp	periventricular nucleus of posterior tuberculum
TS	torus semicularis
Va	valvular cerebelli
VT	ventral thalamus
vTg	ventral tegmentum
X	vagal nerve
Y	yolk sac
