# Supplementary material for: Increased locomotor activity via regulation of GABAergic signalling in foxp2 mutant zebrafish—implications for neurodevelopmental disorders
Source: Transl Psychiatry. 2021 Oct 14;11:529. doi: 10.1038/s41398-021-01651-w (PMC8517032; doi:10.1038/s41398-021-01651-w)
Supplement: Supplementary file 14 — Supplementary Table 5 [file 41398_2021_1651_MOESM14_ESM.pdf]

Gene symbol / ID (Ensembl)	Gene name	Genotype	Mean	Lower limit	Upper limit	P-value
adgrl3.1 / ENSDARG00000061121	adhesion G protein-coupled receptor L3.1					
		+/+	1.0000	0.8610	1.3511	
		+/-	1.0786	0.8735	1.3533	0.5976
		-/-	1.0873	0.9605	1.0412	0.5504
adgrl3(.2) / ENSDARG00000090624	adhesion G protein-coupled receptor L3(.2)					
		+/+	1.0000	0.7926	1.2617	
		+/-	1.4745	0.8762	2.4815	0.3034
		-/-	2.1122	1.4202	3.1413	0.0480
cntnap2a / ENSDARG00000058969	contactin associated protein 2a					
		+/+	1.0000	0.5231	1.9117	
		+/-	0.9473	0.3745	2.3964	0.9380
		-/-	1.3591	0.3321	5.5626	0.7491
cntnap2b / ENSDARG00000074558	contactin associated protein 2b					
		+/+	1.0000	0.7434	1.3452	
		+/-	1.8448	1.6024	2.1238	0.0320
		-/-	1.8035	1.5166	2.1446	0.0410
dusp6 / ENSDARG00000070914	dual specificity phosphatase 6					
		+/+	1.0000	0.8599	1.1630	
		+/-	1.2848	1.0960	1.5060	0.1188
		-/-	1.4903	1.1367	1.9540	0.0898
foxp1a / ENSDARG00000004843	forkhead box P1a					
		+/+	1.0000	0.9120	1.0965	
		+/-	1.0907	0.9314	1.2773	0.4570
		-/-	1.5366	1.2419	1.9011	0.0327
foxp1b / ENSDARG00000014181	forkhead box P1b					
		+/+	1.0000	0.8167	1.2244	
		+/-	1.0581	0.9188	1.2184	0.7123
		-/-	1.2072	0.9285	1.5696	0.3810
foxp2 / ENSDARG00000005453	forkhead box P2					
		+/+	1.0000	0.7706	1.2977	
		+/-	0.5842	0.5468	0.6241	0.0257
		-/-	0.3410	0.2046	0.5684	0.0314
gad1a / ENSDARG00000093411	glutamate decarboxylase 1a					
		+/+	1.0000	0.8081	1.2374	
		+/-	1.1613	1.0548	1.2785	0.3299
		-/-	0.9543	0.5246	1.7361	0.9048
gad1b / ENSDARG00000027419	glutamate decarboxylase 1b					
		+/+	1.0000	0.9736	1.0271	
		+/-	0.8777	0.8381	0.9192	0.0133
		-/-	1.0470	0.7766	1.4115	0.8041
gad2 / ENSDARG00000015537	glutamate decarboxylase 2					
		+/+	1.0000	0.8149	1.2272	
		+/-	1.3036	1.2910	1.3164	0.0885
		-/-	1.1004	0.7116	1.7015	0.7481
grm3 / ENSDARG00000031712	glutamate receptor, metabotropic 3					
		+/+	1.0000	0.7406	1.3503	
		+/-	0.9616	0.8725	1.0598	0.8402
		-/-	1.1090	0.7275	1.6905	0.7467
lrrn1 / ENSDARG00000060115	leucine rich repeat neuronal 1					
		+/+	1.0000	0.9295	1.0758	
		+/-	1.0356	0.9252	1.1593	0.6751
		-/-	1.0123	0.8856	1.1571	0.8962
mef2ca / ENSDARG00000029764	myocyte enhancer factor 2ca					
		+/+	1.0000	0.8500	1.1764	
		+/-	1.4037	1.0165	1.9384	0.1794
		-/-	1.5511	1.1090	2.1695	0.1110
mef2cb / ENSDARG00000009418	myocyte enhancer factor 2cb					
		+/+	1.0000	0.8606	1.1620	
		+/-	1.5804	1.2971	1.9254	0.0331
		-/-	1.5937	1.2113	2.0969	0.0613
ntrk2b / ENSDARG00000098511	neurotrophic tyrosine kinase, receptor, type 2b					
		+/+	1.0000	0.7235	1.3822	
		+/-	1.2195	1.0197	1.4583	0.4053
		-/-	1.3931	0.8530	2.2752	0.3839
pcdh7a / ENSDARG00000078898	protocadherin 7a					
		+/+	1.0000	0.7146	1.3993	
		+/-	1.1458	0.7745	1.6952	0.6714
		-/-	1.8968	1.0962	3.2821	0.1597
pcdh7b / ENSDARG00000060610	protocadherin 7ab					
		+/+	1.0000	0.7713	1.2964	
		+/-	0.9461	0.8512	1.0516	0.7493
		-/-	1.0257	0.7941	1.3247	0.9099
ppp1r1b / ENSDARG00000076280	protein phosphatase 1, regulatory (inhibitor) subunit 1B					
		+/+	1.0000	0.7593	1.3169	
		+/-	1.4334	1.0936	1.8789	0.1815
		-/-	1.3813	1.1286	1.6906	0.1767
sema6d / ENSDARG00000002748	semaphorin 6D					
		+/+	1.0000	0.8385	1.1926	
		+/-	1.0700	0.9111	1.2567	0.6488
		-/-	1.2786	0.9445	1.7309	0.2912
slitrk2 / ENSDARG00000006636	SLIT and NTRK-like family, member 2					
		+/+	1.0000	0.5652	1.7694	
		+/-	1.8939	1.5606	2.2983	0.1403
		-/-	2.1248	1.3995	3.2259	0.1386
