# Supplementary material for: Increased locomotor activity via regulation of GABAergic signalling in foxp2 mutant zebrafish—implications for neurodevelopmental disorders
Source: Transl Psychiatry. 2021 Oct 14;11:529. doi: 10.1038/s41398-021-01651-w (PMC8517032; doi:10.1038/s41398-021-01651-w)
Supplement: Supplementary file 6 — Supplementary Figure 4 [file 41398_2021_1651_MOESM6_ESM.pdf]

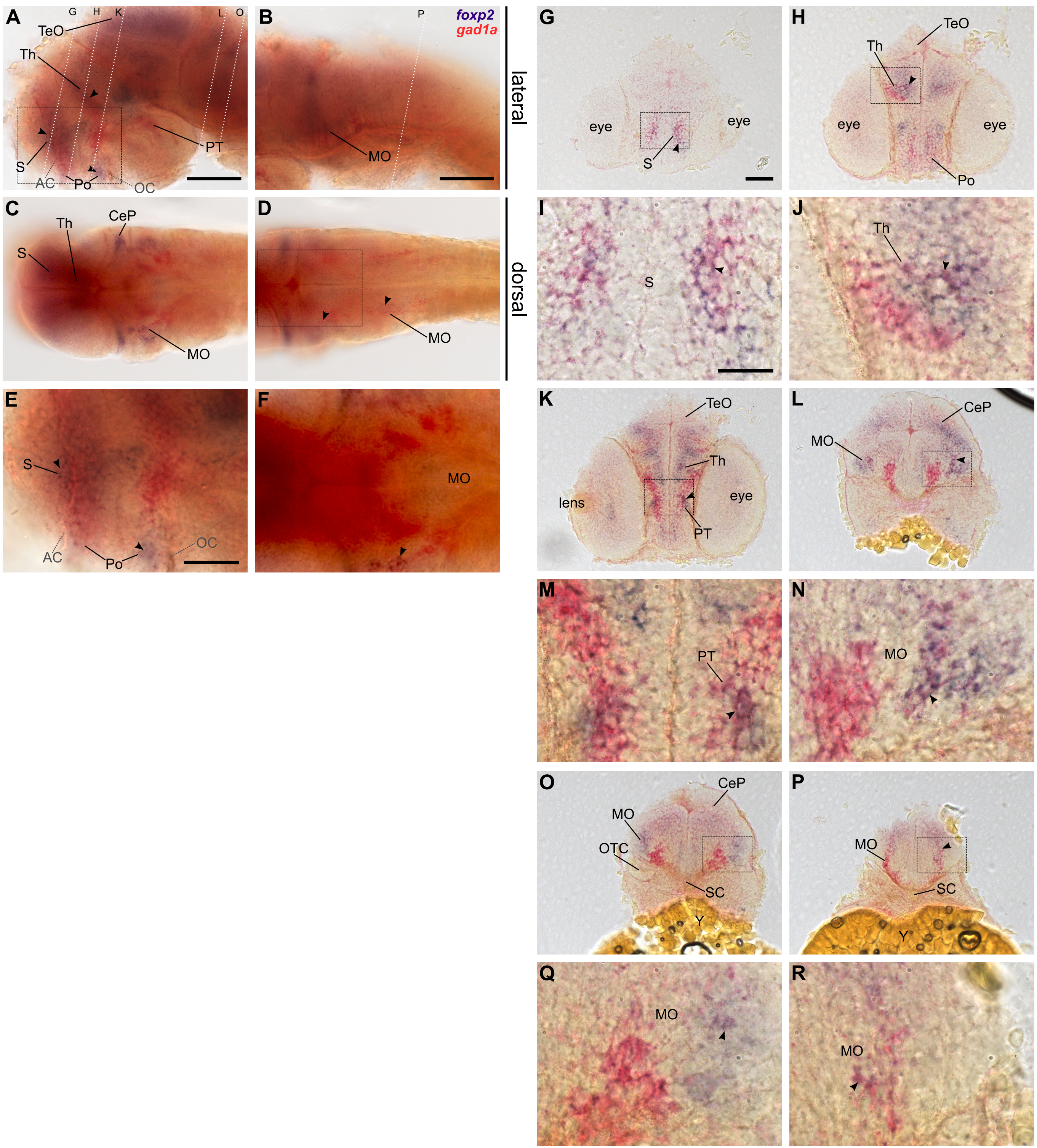

A
B
C
D
E
F
G
H
I
J
K
L
M
N
O
P
Q
R
foxp2
gad1a
lateral
dorsal
TeO
Th
S
AC
Po
OC
PT
MO
CeP
eye
lens
OTC
SC
Y
